# Supplementary material for: Plasmid-Mediated Spread of Carbapenem Resistance in Enterobacterales: A Three-Year Genome-Based Survey
Source: Antibiotics (Basel). 2024 Jul 23;13(8):682. doi: 10.3390/antibiotics13080682 (PMC11350871; doi:10.3390/antibiotics13080682)
Supplement: Supplementary file 1 [file antibiotics-13-00682-s001.zip › antibiotics-3090148-supplementary.pdf]

Supplementary Materials

Titel: Plasmid-Mediated Spread of Carbapenem Resistance in *Enterobacterales*: A Three-Year Genome-Based Survey

Authors: Yancheng Yao [1,2,*], Can Imirzalioglu[1,2], Linda Falgenhauer[1,3,4], Jane Falgenhauer[1,2], Petra Heinmüller[4], the SurvCARE Hesse Working Group[#,], Eugen Domann [1,2,3,*], and Trinad Chakraborty[1,2]

Members of the SurvCARE Hesse Working Group are listed in the acknowledgements section

*, authors for correspondence: yancheng.yao@mikrobio.med.uni-giessen.de or eugen.domann@mikrobio.med.uni-giessen.de. Both authors contributed equally.

Author affiliations:

[1]Institute of Medical Microbiology, Justus Liebig University Giessen, Schubertstrasse 81, 35392 Giessen, Germany

[2]German Center for Infection Research (DZIF), Partner Site Giessen-Marburg-Langen

[3]Institute for Hygiene and Environmental Medicine, Justus Liebig University Giessen, Schubertstrasse 81, 35392 Giessen, Germany

[4]Hessisches Landesamt für Gesundheit und Pflege (HLfGP), Dillenburg, Germany

Supplementary Table S1: Meta data of the bacterial isolates

Isolate	Organism	Sam-pling Year	Source	Gender (1 = male; 0= female); 9= environment	Age (y)	Hospital ID
10302a-17	*K. pneumoniae*	2017	Pharynx mucosa	1	55	
10302b-17	*K. pneumoniae*	2017	Pharynx mucosa	1	55	
10304-17	*K. pneumoniae*	2017	Nasal mucosa	0	63	
10306-17	*K. pneumoniae*	2017	Rectal /stool swab	0	63	
2110128687	*Pseudomonas aeruginosa*	2018	Urine /Catheter urine	1	80	3511
2110129324	*Pseudomonas aeruginosa*	2019	Tracheal secretions	0	83	3511
AF-147492	*Enterobacter xiangfangensis*	2019	unknown			3380
bk17021853	*S. marcescens*	2017	Blood culture	1	85	3770
bk18035705	*C. freundii*	2018	Blood culture	0	79	3770
Entero22	*Enterobacter xiangfangensis*	2019	unknown			3380
Entero23	*Enterobacter xiangfangensis*	2019	unknown			3380
NRZ_29912	*C. freundii*	2016	Rectal /stool swab	0	72	3331
NRZ-16960	*K. pneumoniae*	2014	Nose throat swab	1	59	3841
NRZ-17079	*K. pneumoniae*	2014	Rectal /stool swab	1	61	3841
NRZ-17685	*K. pneumoniae*	2015	Urine /Catheter urine	1	46	3620
NRZ-17710	*K. pneumoniae*	2015	Nose throat swab	0	82	3841
NRZ-17768	*K. pneumoniae*	2015	Liquor	0	70	3841
NRZ-18377	*K. pneumoniae*	2014	Tracheal secretions	1	62	3841
NRZ-24454	*K. pneumoniae*	2016	Urine /Catheter urine	1	72	3711
NRZ-24773	*K. pneumoniae*	2016	Nose throat swab	1	34	3300
NRZ-24797	*K. pneumoniae*	2016	Urine /Catheter urine	1	74	3711
NRZ-26443	*K. pneumoniae*	2016	Rectal /stool swab	0	73	3331
NRZ-26447	*K. pneumoniae*	2016	Wound swab	0	80	3430
NRZ-27806	*K. pneumoniae*	2016	Urethral swab	1	82	3000
NRZ-28673	*K. pneumoniae*	2016	Urine /Catheter urine	0	75	3820
NRZ-29298	*K. pneumoniae*	2016	Rectal /stool swab	1	68	3131
NRZ-29911a	*E. coli*	2016	Rectal /stool swab	0	72	3331
NRZ-30046	*k. pneumoniae*	2016	Rectal /stool swab	0	77	3331
NRZ-30817	*K. pneumoniae*	2016	Anal swab	0	47	3300
NRZ-31125	*k. pneumoniae*	2016	Rectal /stool swab	0	72	3331
NRZ-31131	*C. freundii*	2016	Rectal /stool swab	1	75	3331
NRZ-31836	*C. freundii*	2017	Rectal /stool swab	1	72	3331
NRZ-32009	*E. coli*	2017	Rectal /stool swab	1	72	3331
NRZ-32439	*K. pneumoniae*	2017	Urine /Catheter urine	1	82	3000)
NRZ-32446	*E. coli*	2017	Rectal /stool swab	1	72	3331
NRZ-32813	*Pseudomonas aeruginosa*	2017	Tracheal secretions	1	54	3521
NRZ-33225	*K. pneumoniae*	2017	Blood culture	1	68	3921
NRZ-33411	*E. coli*	2017	Anal swab	0	68	3001
NRZ-33607	*K. pneumoniae*	2017	Lumbar spine subcutaneously	0	68	3201
NRZ-33665	*K. variicola*	2017	Rectal /stool swab	1	81	3331
NRZ-33707	*K. pneumoniae*	2017	Urine /Catheter urine	1	28	3351
NRZ-33707-1	*K. pneumoniae*	2017	Urine /Catheter urine	1	28	3351
NRZ-33707-2	*K. pneumoniae*	2017	Urine /Catheter urine		28	3351
NRZ-33784	*Pseudomonas aeruginosa*	2017	Rectal /stool swab	1	61	3521
NRZ-33852	*S. marcescens*	2017	Tracheal secretions	1	68	3001
NRZ-33910	*E. coli*	2018	unknown			3331
NRZ-33946	*K. pneumoniae*	2017	Anal swab	1	72	3051
NRZ-34952	*Pseudomonas aeruginosa*	2017	Rectal /stool swab	1	29	3521
NRZ-35373	*E. coli*	2017	Anal swab	1	86	3001
NRZ-36109	*K. michiganensis*	2017	Rectal /stool swab	1	74	3651
NRZ-36387	*K. aerogenes*	2018	unknown			3331
NRZ-36455	*K. michiganensis*	2017	Perianal swab	1	84	3651
NRZ-36521	*K. michiganensis*	2017	Nose throat swab	1	64	3001
NRZ-36532	*S. marcescens*	2017	Tracheal secretions	1	82	3001
NRZ-36825	*K. michiganensis*	2017	Rectal /stool swab	0	80	3690
NRZ-36826	*K. michiganensis*	2017	Rectal /stool swab	1	66	3501
NRZ-36855	*K. pneumoniae*	2017	Anal swab	0	18	3451
NRZ-37119	*C. freundii*	2017	Swab screening	1	64	3001

NRZ-37213	*K. pneumoniae*	2017	Urine /Catheter urine	0	74	3900
NRZ-37361	*k. pneumoniae*	2018	Blood culture	1	86	3331
NRZ-37961	*K. pneumoniae*	2017	Anal swab	0	65	3300
NRZ-37969	*C. freundii*	2017	Rectal /stool swab	1	73	3331
NRZ-38150	*K. pneumoniae*	2017	Rectal /stool swab	1	19	3430
NRZ-38364	*E. cloacae*	2017	Rectal /stool swab	0	28	3001
NRZ-38696	*K. pneumoniae*	2017	Urine /Catheter urine	0	87	3000
NRZ-38718	*C. freundii*	2017	Rectal /stool swab	0	82	3331
NRZ-38892	*K. pneumoniae*	2017	Swab screening	1	67	3300
NRZ-38941	*K. pneumoniae*	2018	unknown			3331
NRZ-38954	*C. freundii*	2017	Rectal /stool swab	1	59	3331
NRZ-39678	*K. pneumoniae*	2018	Urine /Catheter urine	0	79	3300
NRZ-39890	*E. coli*	2018	Swab (Screening)	1	75	3400
NRZ-40165	*K. pneumoniae*	2018	Urine /Catheter urine	1	81	3900
NRZ-40345	*K. pneumoniae*	2018	Urine /Catheter urine	1	25	3620
NRZ-40742	*K. pneumoniae*	2018	Swab screening	1	77	3051
NRZ-40869	*K. pneumoniae*	2018	Bronchial secretions/BAL	1	18	3620
NRZ-41277	*K. pneumoniae*	2018	Catheter urine	1	86	3331
NRZ-41473	*K. michiganensis*	2018	Rectal /stool swab	1	82	3651
NRZ-41623	*K. pneumoniae*	2018	Unknown	0	83	3331
NRZ-41658	*S. marcescens*	2018	Swab	1	56	3001
NRZ-41716	*K. pneumoniae*	2018	Urine /Catheter urine	0	82	3530
NRZ-41724	*Raoultella ornithinolytica*	2018	Rectal /stool swab	1	73	3331
NRZ-42446	*K. pneumoniae*	2018	Anal swab	1	82	3550
NRZ-42581	*K. pneumoniae*	2018	Anal swab	1	24	3550
NRZ-43499	*K. pneumoniae*	2018	Urine /Catheter urine	1	80	3300
NRZ-43575	*E. coli*	2018	Anal swab	0	63	3400
NRZ-45112	*K. pneumoniae*	2018	Urine	1	61	3900
NRZ-45233	*C. freundii*	2018	Urine /Catheter urine	0	89	3000
sc17223997	*E. cloacae*	2017	Rectal /stool swab	0	48	3770
sc17225761	*E. coli*	2017	Nose throat swab	1	51	3770
sc17236944	*E. coli*	2017	Rectal /stool swab	0	55	3770
sc17238571	*C. freundii*	2017	Rectal /stool swab	1	52	3770
sc17259839	*E. cloacae*	2017	Rectal /stool swab	0	70	3770
sc17262611	*k. pneumoniae*	2017	Rectal /stool swab	0	0	3770
sc17266060	*E. cloacae*	2017	Rectal /stool swab	0	0	3770
sc17286987	*C. freundii*	2017	Rectal /stool swab	1	46	3770
sc17288868	*K. pneumoniae*	2017	Rectal /stool swab	1	25	3770
sc17290012	*k. pneumoniae*	2017	Rectal /stool swab	0	63	3770
sc17290660	*k. pneumoniae*	2017	Rectal /stool swab	1	16	3770
sc17292733	*k. pneumoniae*	2017	Nose throat swab	0	77	3770
sc17294875	*E. cloacae*	2017	Nose throat swab	1	0	3770
sc17304042	*E. coli*	2017	Rectal /stool swab	1	86	3770
sc17312208	*K. pneumoniae*	2017	Nose throat swab	1	65	3770
sc17326118	*K. pneumoniae*	2017	Rectal /stool swab	1	85	3770
sc17326834	*k. pneumoniae*	2017	Rectal /stool swab	1	47	3770
sc18328822	*K. pneumoniae*	2018	Rectal /stool swab	1	81	3770
sc18330209	*K. pneumoniae*	2018	Rectal /stool swab	1	61	3770
sc18353261	*K. pneumoniae*	2018	Rectal /stool swab	1	57	3770
sc18364826	*C. freundii*	2018	Rectal /stool swab	1	63	3770
sc18379832	*K. aerogenes*	2018	Nose throat swab	1	67	3770
sc18383857	*K. pneumoniae*	2018	Rectal /stool swab	1	78	3770
sc18393485	*E. coli*	2018	Rectal /stool swab	0	65	3770
sc18400719	*Enterobacter xiangfangensis*	2018	Nose throat swab	1	80	3770
sc18400986	*Enterobacter xiangfangensis*	2018	Rectal /stool swab	1	32	3770
sc18402389	*Enterobacter xiangfangensis*	2018	Rectal /stool swab	0	0	3770
sc18407397	*C. portucalensis*	2018	Rectal /stool swab	0	59	3770
sc18408856	*E. coli*	2018	Rectal /stool swab	0	28	3770
sc18411631	*E. coli*	2018	Rectal /stool swab	0	36	3770
sc18412584	*C. freundii*	2018	Rectal /stool swab	1	68	3770
sc18412783	*C. portucalensis*	2018	Rectal /stool swab	1	68	3770
sc18415124	*K. pneumoniae*	2018	Rectal /stool swab	1	89	3770
sc19427838	*Enterobacter kobei*	2019	Rectal /stool swab	0	53	3770
sc19434657	*Enterobacter xiangfangensis*	2019	Rectal /stool swab	0	80	3770
sc19437368	*k. pneumoniae*	2019	Tracheal secretions	1	18	3770
sc19437721	*E. coli*	2019	Nose throat swab	0	30	3770
sc19441634	*K. aerogenes*	2019	Rectal /stool swab	1	0	3770

sc19443197	*K. aerogenes*	2019	Rectal /stool swab	0	67	3770
sc19443240	*K. aerogenes*	2019	Rectal /stool swab	1	36	3770
sc19443521-1	*K. pneumoniae*	2019	Rectal /stool swab	1	69	3770
sc19443521-2	*K. pneumoniae*	2019	Rectal /stool swab	1	69	3770
sc19444294-1	*C. freundii*	2019	Nose throat swab	1	80	3770
sc19444656	*K. aerogenes*	2019	Rectal /stool swab	1	61	3770
sc19445662	*K. aerogenes*	2019	Rectal /stool swab	1	53	3770
sc19446287	*C. freundii*	2019	Rectal /stool swab	0	93	3770
sc19460931	*E. cloacae*	2019	Rectal /stool swab	0	0	3770
sp17013730	*K. aerogenes*	2017	Tracheal secretions	1	62	3770
sp17015437	*K. pneumoniae*	2017	Sputum	1	62	3770
sp17017045	*A. baumannii*	2017	Sputum	1	76	3770
sp18021199	*E. coli*	2018	Tracheal secretions	0	49	3770
sp19025051	*K. pneumoniae*	2019	Sputum	1	80	3770
st17007067	*K. pneumoniae*	2017	Stool	1	.	3770
Survca012	*K. pneumoniae*	2017	Rectal /stool swab	1	19	3430
Survca013	*K. variicola*	2017	Rectal /stool swab	0	47	3430
Survcare Ex01	*K. pneumoniae*	2017	Rectal /stool swab	1	76	2000
Survcare Ex04	*K. pneumoniae*	2017	Rectal /stool swab	0	71	3620
Survcare Ex05	*K. pneumoniae*	2017	unknown	1	76	3600
Survcare Ex06	*K. pneumoniae*	2017	unknown	1	80	3600
Survcare Ex07	*K. pneumoniae*	2017	Rectal /stool swab	1	80	3600
Survcare Ex08	*K. pneumoniae*	2017	unknown	1	91	3600
Survcare Ex09	*K. pneumoniae*	2017	Throat swab	1	73	3600
Survcare Ex10	*K. pneumoniae*	2017	Urine /Catheter urine	0	79	3600
Survcare Ex11	*K. pneumoniae*	2017	Rectal /stool swab	0	81	3031
Survcare Ex12	*K. pneumoniae*	2017	Hospital environment	9		3600
Survcare002	*K. aerogenes*	2017	Rectal /stool swab	1	81	3470
Survcare003	*A. baumannii*	2017	Rectal /stool swab	0	31	3560
Survcare004	*A. baumannii*	2017	intra-operative material intra-articular	1	34	3560
Survcare005	*A. baumannii*	2017	wound swab	1	73	3560
Survcare006	*A. baumannii*	2017	Rectal /stool swab	1	58	3560
Survcare007	*A. baumannii*	2017	Nose throat swab	1	51	3560
Survcare008	*K. pneumoniae*	2017	Rectal /stool swab	1	77	3600
Survcare009	*K. pneumoniae*	2017	Rectal /stool swab	0	66	3600
Survcare011	*K. pneumoniae*	2017	Urine /Catheter urine	0	73	3600
Survcare014	*A. baumannii*	2017	Rectal /stool swab	1	70	3430
Survcare015	*A. baumannii*	2017	Sputum	0	50	3560
Survcare016	*A. baumannii*	2017	unknown	1	51	3640
Survcare017	*A. baumannii*	2017	Rectal /stool swab	1	65	3640
Survcare018	*K. pneumoniae*	2017	Rectal /stool swab	0	85	3430
Survcare019	*A. baumannii*	2017	Rectal /stool swab	0	70	3430
Survcare020	*K. pneumoniae*	2017	Rectal /stool swab	0	70	3430
Survcare021	*K. pneumoniae*	2017	Urine /Catheter urine	0	64	3600
Survcare022	*K. pneumoniae*	2017	Rectal /stool swab	0	75	3900
Survcare023	*K. pneumoniae*	2017	Throat swab	0	74	3900
Survcare024	*K. pneumoniae*	2018	Hospital environment	9		3900
Survcare025	*K. pneumoniae*	2018	Stool	0	70	3430
Survcare027	*K. pneumoniae*	2018	Rectal /stool swab	1	79	3560
Survcare033	*K. pneumoniae*	2018	Urine /Catheter urine	1	74	3560
Survcare034	*K. pneumoniae*	2018	Hospital environment	9		3560
Survcare035	*K. pneumoniae*	2018	Urine /Catheter urine	0	75	3560
Survcare036	*C. freundii*	2018	Rectal /stool swab	1	63	3560
Survcare037	*K. pneumoniae*	2018	Rectal /stool swab	1	73	3560
Survcare038	*K. pneumoniae*	2018	Rectal /stool swab	1	76	3560
Survcare039	*K. pneumoniae*	2018	Urine /Catheter urine	1	60	3560
Survcare040	*K. pneumoniae*	2017	Rectal /stool swab	1	59	3560
Survcare041	*K. pneumoniae*	2018	Skin swab	1	77	3560
Survcare042	*K. pneumoniae*	2018	Rectal /stool swab	1	81	3560
Survcare043	*E. coli*	2018	Ascites	0	67	3430
Survcare044	*K. pneumoniae*	2017	Urine /Catheter urine	0	83	3561
Survcare045	*E. coli*	2018	Skin swab	0	65	3430
Survcare046	*K. pneumoniae*	2018	Rectal /stool swab	0	62	3560
Survcare047	*K. pneumoniae*	2017	Urine /Catheter urine	1	28	3750
Survcare048	*A. baumannii*	2017	Urine /Catheter urine	1	68	3750
Survcare049	*A. baumannii*	2017	Tracheal secretions	1	65	3750

Survcare050	*C. freundii*	2017	Abscess swab	0	44	3750
Survcare051-1	*K. oxytoca*	2017	unknown		84	3161
Survcare051-2	*A. baumannii*	2017	Rectal /stool swab	0	84	3161
Survcare052	*E. coli*	2017	Rectal /stool swab	0	59	3161
Survcare053-1	*E. coli*	2017	Rectal /stool swab	1	71	3161
Survcare053-2	*K. pneumoniae*	2017	Rectal /stool swab	1	71	3161
Survcare053-3	*A. baumannii*	2017	Rectal /stool swab	1	71	3161
Survcare055	*K. pneumoniae*	2018	Rectal /stool swab	1	84	3750
Survcare056	*K. pneumoniae*	2018	Rectal /stool swab	1	77	3161
Survcare057	*A. baumannii*	2017	Tracheal secretions	1	73	3750
Survcare058	*E. coli*	2017	Rectal /stool swab	1	83	3750
Survcare059	*A. baumannii*	2017	Groin swab	1	55	3750
Survcare060	*k. oxytoca*	2017	Wound swab foot	1	80	3750
Survcare061-1	*S. marcescens*	2017	Wound swab foot	1	80	3750
Survcare061-2	*K. oxytoca*	2017	Wound swab foot	1	80	3750
Survcare061-3	*A. baumannii*	2017	Wound swab foot	1	80	3750
Survcare062-1	*A. baumannii*	2017	Wound swab foot	1	80	3750
Survcare062-2	*k. oxytoca*	2017	unknown	1	80	3750
Survcare063-1	*E. coli*	2017	Swab Leiste	0	72	3750
Survcare063-2	*k. oxytoca*	2017	unknown	0	72	3750
Survcare063-3	*A. baumannii*	2017	unknown	0	72	3750
Survcare064	*A. baumannii*	2017	Wound swab leg	1	70	3750
Survcare065	*K. pneumoniae*	2018	Skin swab	1	41	3560
Survcare066-1	*A. baumannii*	2016	Tracheal secretions	1	64	3750
Survcare066-2	*k. oxytoca*	2016	Tracheal secretions	1	64	3750
Survcare067-1	*A. baumannii*	2015	Throat swab	1	76	3750
Survcare067-2	*k. oxytoca*	2015	unknown	1	76	3750
Survcare068-1	*A. baumannii*	2016	Bronchial secretions/BAL	1	52	3750
Survcare068-2	*k. oxytoca*	2016	BAL	1	52	3750
Survcare069	*E. coli*	2016	Gallbladder	1	83	3161
Survcare070	*K. pneumoniae*	2015	Rectal /stool swab	1	84	3161
Survcare071	*K. pneumoniae*	2016	Urine /Catheter urine	0	55	3161
Survcare072	*E. cloacae*	2016	Rectal /stool swab	1	76	3161
Survcare074	*k. oxytoca*	2016	Urine /Catheter urine	1	77	3750
Survcare075	*K. pneumoniae*	2016	Rectal /stool swab	1	78	3750
Survcare076	*K. pneumoniae*	2016	Wound swab	1	81	3750
Survcare077	*E. coli*	2016	Rectal /stool swab	1	48	3750
Survcare078	*K. variicola*	2016	Urine /Catheter urine	0	71	3750
Survcare079	*E. cloacae*	2016	Urine /Catheter urine	0	89	3750
Survcare080	*C. freundii*	2016	Rectal /stool swab	1	69	3750
Survcare081	*E. coli*	2016	Urine /Catheter urine	1	69	3750
Survcare082	*K. pneumoniae*	2016	Urine /Catheter urine	1	69	3750
Survcare083-1	*C. freundii*	2016	Groin swab	1	74	3750
Survcare083-2	*k. oxytoca*	2016	Groin swab		74	3750
Survcare084	*E. cloacae*	2016	Pleural cavity	1	78	3750
Survcare085	*Raoultella planticola*	2018	Rectal /stool swab	1	67	3651
Survcare086	*Raoultella planticola*	2018	Rectal /stool swab	1	72	3651
SurvCare087	*K. michiganensis*	2017	Urine /Catheter urine	0	79	3880
Survcare088	*A. baumannii*	2018	Rectal /stool swab	1	84	3750
Survcare089	*A. baumannii*	2018	Rectal /stool swab	1	75	3161
Survcare090	*K. pneumoniae*	2018	Ascites	1	82	3810
Survcare091	*K. pneumoniae*	2018	Blood culture	1	70	3810
Survcare092	*C. freundii*	2018	Rectal /stool swab	1	75	3331
Survcare094	*E. coli*	2018	Wound swab (Skin necrosis)	1	73	3161
Survcare095	*E. cloacae*	2018	Blood culture	1	62	3430
Survcare096	*E. cloacae*	2018	Wound swab knee	1	69	3161
Survcare097	*K. variicola*	2018	Rectal /stool swab	1	75	3331
Survcare098	*S. marcescens*	2018	Bronchial secretions/BAL	0	31	3001
Survcare099	*S. marcescens*	2018	Urethral Swab	1	56	3001
Survcare100	*S. marcescens*	2017	Bronchial secretions/BAL	1	68	3001
Survcare101	*S. marcescens*	2018	Bronchial secretions/BAL	1	61	3001
Survcare102	*S. marcescens*	2018	Rectal /stool swab	1	72	3001
Survcare103	*E. coli*	2018	Rectal /stool swab	1	75	3331

Survcare104	*E. coli*	2018	Rectal /stool swab	1	75	3331
Survcare105	*K. pneumoniae*	2018	Rectal /stool swab	1	68	3331
Survcare107	*K. pneumoniae*	2018	Rectal /stool swab	1	87	3331
Survcare108	*K. pneumoniae*	2018	Urine /Catheter urine	0	87	3300
Survcare109	*K. pneumoniae*	2018	TK	0	67	3300
Survcare111	*E. coli*	2018	Rectal /stool swab	1	71	3430
Survcare112	*A. baumannii*	2018	Rectal /stool swab	0	78	3161
Survcare113	*K. pneumoniae*	2018	Rectal /stool swab	0	78	3161
Survcare114	*E. coli*	2018	Urine /Catheter urine	0	28	3521
Survcare115	*k. pneumoniae*	2018	Rectal /stool swab	1	74	3430
Survcare116	*A. baumannii*	2018	Urinary catheter	0	69	3161
Survcare117	*K. pneumoniae*	2018	PEG swab	0	67	3300
Survcare118	*K. pneumoniae*	2018	Tracheal secretions	0	67	3300
Survcare119	*E. coli*	2018	Urine /Catheter urine	0	23	3000
Survcare120	*C. freundii*	2018	Abdominal swab	1	61	3560
Survcare121	*E. coli*	2018	Urine /Catheter urine	1	77	3750
Survcare122	*A. baumannii*	2018	Urine /Catheter urine	0	74	3000
Survcare123	*K. pneumoniae*	2018	Tracheal secretions	1	71	3430
Survcare124	*K. michiganensis*	2018	Rectal /stool swab	0	67	3001
Survcare125	*E. coli*	2018	Rectal /stool swab	0	56	3331
Survcare126	*K. michiganensis*	2018	Rectal /stool swab	1	70	3001
Survcare127	*K. pneumoniae*	2018	Rectal /stool swab	0	54	3001
Survcare128	*Pseudomonas aeruginosa*	2018	Tracheal secretion from tracheostoma	1	26	3121
Survcare129	*E. coli*	2018	Rectal /stool swab	1	72	3001
Survcare130	*E. coli*	2018	Bile	1	70	3941
Survcare131	*K. pneumoniae*	2018	Bile	0	70	3560
Survcare132	*C. freundii*	2018	Rectal /stool swab	1	70	3430
Survcare133	*E. coli*	2018	Rectal /stool swab	1	68	3230
Survcare134	*E. coli*	2018	Rectal /stool swab	1	68	3230
Survcare135	*E. cloacae*	2018	Rectal /stool swab	0	76	3430
Survcare137	*C. freundii*	2018	Rectal /stool swab	0	66	3750
Survcare138	*A. baumannii*	2018	Rectal /stool swab	0	66	3750
Survcare139	*K. pneumoniae*	2018	Groin swab	1	76	3750
Survcare140	*E. coli*	2018	Serom re UB	0	52	3750
Survcare141	*A. baumannii*	2018	Urine /Catheter urine	0	26	3750
Survcare142	*K. pneumoniae*	2018	Urine /Catheter urine	0	78	3750
Survcare143	*Proteus mirabilis*	2018	Urine /Catheter urine	1	89	3331
Survcare145	*K. pneumoniae*	2018	Urine /Catheter urine	1	59	3820
Survcare146	*E. cloacae*	2018	Rectal /stool swab	1	75	3521
Survcare148	*A. baumannii*	2018	Nose throat swab	1	66	3380
Survcare149	*K. pneumoniae*	2018	Urine /Catheter urine	1	82	3400
Survcare150	*C. freundii*	2018	Rectal /stool swab	1	68	3941
Survcare151	*E. cloacae*	2018	Wound swab	0	42	3631
Survcare153	*K. pneumoniae*	2018	Rectal /stool swab	0	58	3161
Survcare154	*C. freundii*	2018	Rectal /stool swab	1	58	3520
Survcare155	*Pseudomonas aeruginosa*	2018	Urine /Catheter urine	1	58	3860
Survcare156	*Pseudomonas aeruginosa*	2018	Urine /Catheter urine	0	71	3000
Survcare157	*Pseudomonas aeruginosa*	2018	Urine /Catheter urine	1	70	3000
Survcare158	*Pseudomonas aeruginosa*	2018	Urine /Catheter urine	0	65	3281
Survcare159	*Pseudomonas aeruginosa*	2018	Urine /Catheter urine	1	60	3120
Survcare160	*C. freundii*	2018	Rectal /stool swab	1	77	3331
Survcare161	*C. freundii*	2018	Urine /Catheter urine	1	75	4000
Survcare162	*C. freundii*	2018	Urethral swab	1	75	4000
Survcare163	*C. freundii*	2018	Intra-abdominale	1	61	4000
Survcare164	*K. pneumoniae*	2018	Rectal /stool swab	1	88	3331
Survcare165	*C. freundii*	2018	Urine /Catheter urine	1	65	4000
Survcare166	*Morganella morganii*	2018	Stuhl	0	43	3941
Survcare167	*K. aerogenes*	2018	Rectal /stool swab	1	0	3521
Survcare168	*E. coli*	2018	Rectal /stool swab	1	76	3480
Survcare169	*Enterobacter xiangfangensis*	2018	Anal swab	1	75	3611
Survcare170	*K. pneumoniae*	2018	Urine /Catheter urine	1	86	3750
Survcare171	*K. variicola*	2018	Rectal /stool swab	1	71	3480
Survcare172	*K. pneumoniae*	2018	Urine /Catheter urine	0	75	3001
Survcare173	*K. pneumoniae*	2018	Ulcer foot	1	80	3521
Survcare174	*S. marcescens*	2018	Rectal /stool swab	0	58	3520
Survcare175	*Pseudomonas stutzeri*	2018	Tissue necrose	0	72	3380
Survcare176	*E. coli*	2018	Rectal /stool swab	1	67	3430

Survcare177	*Pseudomonas aeruginosa*	2018	Urine	0	47	3000
Survcare178	*Proteus mirabilis*	2018	Rectal /stool swab	1	66	3430
Survcare179	*K. oxytoca*	2018	Rectal /stool swab	0	64	3430
Survcare180	*K. aerogenes*	2018	Rectal /stool swab	1	67	3430
Survcare182	*Pseudomonas aeruginosa*	2018	Tracheal secretions	1	62	3521
Survcare184	*E. coli*	2018	Rectal /stool swab	0	65	3161
Survcare185	*A. baumannii*	2018	Rectal /stool swab	1	62	3331
Survcare186	*K. pneumoniae*	2018	Rectal /stool swab	1	70	3331
Survcare188	*C. freundii*	2018	Rectal /stool swab	1	19	3521
Survcare189	*K. pneumoniae*	2018	Rectal /stool swab	0	70	3480
Survcare190	*E. coli*	2018	Wound swab	1	80	3611
Survcare191	*K. pneumoniae*	2018	Wound swab	1	80	3611
Survcare192	*Enterobacter xiangfangensis*	2018	Rectal /stool swab	0	74	3161
Survcare193	*K. aerogenes*	2018	Tracheal secretions	1	64	3430
Survcare194	*K. pneumoniae*	2018	Breathing tube	1	75	3430
Survcare202	*K. pneumoniae*	2018	Rectal /stool swab	1	68	3611
Survcare203	*Enterobacter xiangfangensis*	2018	Wound swab	1	80	3611
Survcare204	*K. pneumoniae*	2018	Wound swab	0	75	3830
Survcare212	*E. coli*	2019	Rectal /stool swab	1	56	3331
Survcare213	*A. baumannii*	2019	Wound swab	0	37	3331
Survcare217	*K. pneumoniae*	2019	Abscess pancreatic	0	77	3810
Survcare218	*Enterobacter xiangfangensis*	2019	Rectal /stool swab	0	77	3331
Survcare219	*Enterobacter kobei*	2019	Rectal /stool swab	0	69	3331
Survcare221	*E. coli*	2019	Rectal /stool swab	1	85	3221
Survcare222	*K. pneumoniae*	2019	Rectal /stool swab	0	79	3380
Survcare223	*A. baumannii*	2019	Rectal /stool swab	0	79	3380
Survcare224	*K. aerogenes*	2019	Swab butocks	1	83	3530
Survcare225	*Enterobacter xiangfangensis*	2019	Urine /Catheter urine	0	78	3750
Survcare226	*K. pneumoniae*	2019	Bronchial secretions/BAL	0	74	3161
Survcare227	*S. marcescens*	2019	Rectal /stool swab	1	43	3001
Survcare228	*S. marcescens*	2019	Environmental investigation Siphon in room 1	9		3001
Survcare229	*K. pneumoniae*	2019	Tracheal secretions	1	74	3530
Survcare230	*E. coli*	2019	Rectal /stool swab	1	75	3430
Survcare231	*K. oxytoca*	2019	Rectal /stool swab	1	4	3330
Survcare232	*Enterobacter xiangfangensis*	2019	Rectal /stool swab	1	69	3331
Survcare233	*E. cloacae*	2019	Rectal swab	1	49	3390
Survcare234	*E. coli*	2019	Intra-abdominale	1	78	3820
Survcare235	*C. portucalensis*	2019	Rectal /stool swab	0	80	3331
Survcare236	*E. coli*	2019	Rectal /stool swab	1	69	3331
Survcare237	*K. aerogenes*	2019	Rectal /stool swab	0	82	3331
Survcare238-2	*S. marcescens*	2018	Rectal /stool swab	0	39	3001
Survcare239	*C. freundii*	2019	Rectal /stool swab	1	51	3430
Survcare240	*Cedecea lapagei*	2019	Sputum	1	69	3860
Survcare241	*K. pneumoniae*	2019	Rectal /stool swab	0	77	3331
Survcare242	*C. freundii*	2019	transurethral permanent catheter	1	61	3520
Survcare243	*K. pneumoniae*	2019	Rectal /stool swab	0	54	3001
Survcare244	*E. coli*	2019	Rectal /stool swab	0	29	3001
Survcare245	*K. aerogenes*	2019	Wound swab	1	54	3430
Survcare246	*K. aerogenes*	2019	Rectal /stool swab	1	62	3001
Survcare247	*C. freundii*	2019	Swab perineum	1	76	3060
Survcare248	*K. pneumoniae*	2019	Skin swab	1	83	3640
Survcare249	*K. pneumoniae*	2019	Rectal /stool swab	0	55	3580
Survcare250	*K. pneumoniae*	2019	Rectal /stool swab	0	30	3401
Survcare251	*K. pneumoniae*	2019	Rectal /stool swab	1	69	3750
Survcare252	*C. freundii*	2019	Rectal /stool swab	1	85	3820
Survcare253	*E. coli*	2019	Urine /Catheter urine	0	76	3380
Survcare254	*E. coli*	2019	Rectal /stool swab	1	83	3430
Survcare255	*A. baumannii*	2019	Bronchial secretions/BAL	1	74	3331
Survcare257	*Enterobacter xiangfangensis*	2019	Bronchial secretions/BAL	1	69	3300
Survcare258	*S. marcescens*	2019	Rectal /stool swab	1	68	3001
Survcare260	*K. pneumoniae*	2018	Urine /Catheter urine	0	75	3001
Survcare261	*K. pneumoniae*	2019	Rectal /stool swab	1	63	3300

Survcare262	*E. coli*	2019	Rectal /stool swab	1	63	3300
Survcare263	*K. pneumoniae*	2019	Urine /Catheter urine	0	81	3000
Survcare265	*E. coli*	2019	Bronchial secretions/BAL	1	69	3300
Survcare266	*A. baumannii*	2019	Rectal /stool swab	1	63	3331
Survcare267	*K. pneumoniae*	2019	Rectal /stool swab	1	63	3331
Survcare268	*S. marcescens*	2019	Bronchial secretions/BAL	1	61	3001
Survcare269	*K. pneumoniae*	2019	Rectal /stool swab	1	37	3430
Survcare270	*S. marcescens*	2019	Rectal /stool swab	0	79	3001
Survcare271	*K. pneumoniae*	2019	Urine /Catheter urine	0	77	3480
Survcare272	*A. baumannii*	2019	Rectal /stool swab	1	0	3380
Survcare273	*K. pneumoniae*	2019	Urine /Catheter urine	1	79	3480
Survcare274	*K. aerogenes*	2019	Rectal /stool swab	0	75	3001
Survcare275	*E. coli*	2019	Skin swab	0	65	3380
Survcare276	*K. michiganensis*	2019	Urine /Catheter urine	0	79	3001
Survcare277	*A. baumannii*	2019	Wound swab	1	42	3750
Survcare278	*E. coli*	2019	Rectal /stool swab	1	64	3001
Survcare279	*K. pneumoniae*	2019	Rectal /stool swab	1	79	3720
Survcare280	*K. pneumoniae*	2019	PEG swab	1	38	3300
Survcare281	*S. marcescens*	2019	Environmental sample water No. 5	9		3001
Survcare282	*S. marcescens*	2019	Environmental sample water No. 4	9		3001
Survcare283	*K. aerogenes*	2019	Skin swab	1	53	3520
Survcare284	*K. pneumoniae*	2019	Rectal /stool swab	1	64	3001
Survcare285	*A. baumannii*	2019	Urine /Catheter urine	1	78	3561
Survcare286	*K. pneumoniae*	2019	Nose throat swab	0	74	3380
Survcare287	*K. pneumoniae*	2019	Rectal /stool swab	0	56	3750
Survcare289	*E. coli*	2019	Central venous catheter tip	0	15	3380
Survcare290	*K. pneumoniae*	2019	Nose throat swab	0	65	3380
Survcare291	*E. coli*	2019	Nose throat swab	0	65	3380
Survcare292	*E. coli*	2019	Rectal /stool swab	1	78	3331
Survcare293	*E. coli*	2019	Rectal /stool swab	0	0	3330
Survcare294	*S. marcescens*	2019	Rectal /stool swab	1	72	3001
Survcare295	*E. coli*	2019	Rectal /stool swab	1	80	3820
Survcare296	*E. cloacae*	2019	Blood culture	0	56	3941
Survcare298	*Morganella morganii*	2019	Rectal /stool swab	1	81	3640
Survcare299	*A. baumannii*	2019	Rectal /stool swab	1	83	3670
Survcare300	*K. pneumoniae*	2019	Rectal /stool swab	0	1	3740
Survcare302	*K. oxytoca*	2019	Rectal /stool swab	1	83	3670
Survcare303	*E. coli*	2019	Rectal /stool swab	1	79	3640
Survcare306	*C. freundii*	2019	Rectal /stool swab	1	78	3331
Survcare308	*C. portucalensis*	2019	Rectal /stool swab	1	0	3330
Survcare309	*E. coli*	2019	Rectal /stool swab	1	0	3330
Survcare310	*E. coli*	2019	Rectal /stool swab	0	56	3750
Survcare311	*C. freundii*	2019	Rectal /stool swab	0	79	3001
Survcare312	*K. pneumoniae*	2019	Rectal /stool swab	1	59	3200
Survcare313	*E. coli*	2019	Skin swab	1	66	3461
Survcare314	*C. freundii*	2019	Rectal /stool swab	1	90	3560
Survcare315	*C. freundii*	2019	Rectal /stool swab	1	66	3560
Survcare316	*C. freundii*	2018	Hospital environment	9		3560
Survcare317	*E. coli*	2019	Rectal /stool swab	9		3200
Survcare318	*E. coli*	2019	Urine /Catheter urine	0	74	3380
Survcare319	*E. coli*	2019	Rectal /stool swab	0	63	3001
Survcare320	*C. freundii*	2019	Wound swab foot	1	65	3001
Survcare321	*E. coli*	2019	Urine /Catheter urine	1	81	3000
Survcare323	*A. baumannii*	2019	Rectal /stool swab	0	71	3430
Survcare324	*A. baumannii*	2019	Tracheal secretions	1	59	3430
Survcare325	*K. aerogenes*	2019	Rectal /stool swab	1	64	3001
Survcare326	*K. aerogenes*	2019	Rectal /stool swab	0	67	3300
Survcare327	*E. coli*	2019	Dekubitus swab	0	71	3120
Survcare328	*K. pneumoniae*	2019	Rectal /stool swab	1	40	3300
Survcare329	*E. coli*	2019	Rectal /stool swab	0	82	3300
Survcare330	*A. baumannii*	2019	Bronchial secretions/BAL	1	76	3711
Survcare331	*A. baumannii*	2019	Rectal /stool swab	1	78	3711

Survcare332	*A. baumannii*	2019	Penis swab	1	80	3711
Survcare333	*K. pneumoniae*	2019	Tracheal secretions	0	77	3750
Survcare334	*E. coli*	2019	Rectal /stool swab	1	63	3331
Survcare335	*E. coli*	2019	Gallbladder	1	83	3941
Survcare336	*C. braakii*	2019	Rectal /stool swab	1	74	3331
Survcare337	*E. coli*	2019	Rectal /stool swab	1	74	3331
Survcare338	*K. pneumoniae*	2019	Nose throat swab	1	30	3300
Survcare339	*K. aerogenes*	2019	Tracheal secretions	1	71	3120
Survcare340	*A. baumannii*	2019	Rectal /stool swab	0	78	3331
Survcare341	*E. coli*	2019	Rectal /stool swab	1	73	3331
Survcare342	*K. aerogenes*	2019	Anal swab	0	80	3831
Survcare343	*K. pneumoniae*	2019	Anal swab	1	80	3711
Survcare344	*A. pittii*	2019	Rectal /stool swab	1	61	3820
Survcare346	*Enterobacter xiangfangensis*	2019	Wound swab pancreatic pseudocyst	0	56	3941
Survcare347	*C. koseri*	2019	Rectal /stool swab	1	58	3331
Survcare348	*Enterobacter xiangfangensis*	2019	Rectal /stool swab	0	78	3331
Survcare349	*K. pneumoniae*	2019	Rectal /stool swab	0	65	3331
Survcare350	*E. coli*	2019	Rectal /stool swab	0	65	3331
Survcare351	*K. pneumoniae*	2019	Rectal /stool swab	0	81	3711
Survcare353	*K. pneumoniae*	2019	Urine /Catheter urine	0	87	3820
Survcare354	*K. pneumoniae*	2019	Rectal /stool swab	0	72	3430
Survcare355	*Enterobacter xiangfangensis*	2019	Urine /Catheter urine	1	54	3200
Survcare356	*A. baumannii*	2019	Rectal /stool swab	1	87	3060
Survcare357	*Proteus mirabilis*	2019	Groin swab	0	82	3750
Survcare358	*Enterobacter kobei*	2019	Rectal swab	1	50	3520
Survcare359	*A. baumannii*	2019	Urine /Catheter urine	0	77	3750
Survcare360	*E. coli*	2019	Rectal /stool swab	0	79	3000
Survcare361	*K. pneumoniae*	2019	Rectal /stool swab	0	79	3000
Survcare362	*Enterobacter xiangfangensis*	2019	others	1	56	
Survcare364	*K. pneumoniae*	2019	Rectal /stool swab	1	63	3520
Survcare365	*K. michiganensis*	2019	Rectal /stool swab	1	81	3331
Survcare366	*Enterobacter xiangfangensis*	2019	Rectal /stool swab	1	57	3001
Survcare367	*K. pneumoniae*	2019	Wound swab ureter	0	81	3211
Survcare368	*K. pneumoniae*	2019	Rectal /stool swab	1	73	3161
Survcare369	*E. cloacae*	2019	Rectal /stool swab	0	51	3430
Survcare370	*K. aerogenes*	2019	Tracheal secretions	1	87	3430
Survcare371	*K. michiganensis*	2019	Rectal /stool swab	1	90	3001
Survcare372	*Proteus mirabilis*	2019	Blood culture	1	80	3580
Survcare374	*K. pneumoniae*	2019	Rectal /stool swab	1	71	3331
Survcare375	*K. pneumoniae*	2019	Rectal /stool swab	1		3331
Survcare376	*K. pneumoniae*	2019	Rectal /stool swab	1	74	3331
Survcare377	*E. coli*	2019	Rectal /stool swab	1	30	3921
Survcare378	*E. coli*	2019	Urine /Catheter urine	0	29	3921
Survcare379	*E. coli*	2019	Rectal /stool swab	0	56	3921
Survcare380	*E. coli*	2019	Rectal /stool swab	1	67	3921
Survcare381	*E. coli*	2019	Urikult	0	48	3000
Survcare382	*E. coli*	2019	Vaginal Swab	0	29	3521
Survcare383	*E. coli*	2019	Nose throat swab	0	0	3521
Survcare384	*E. coli*	2019	Nose throat swab	0	0	3521
Survcare385	*E. coli*	2019	Skin swab	1	42	3430
Survcare386	*E. coli*	2019	Rectal /stool swab	0	76	3750
Survcare387	*E. coli*	2019	Rectal /stool swab	0	64	3001
Survcare388	*E. coli*	2019	Rectal /stool swab	0	64	3520
Survcare389	*E. coli*	2019	Rectal /stool swab	0	0	3521
Survcare390	*E. coli*	2019	Rectal /stool swab	0	0	3521
Survcare391	*A. baumannii*	2019	Tracheal secretions	1	38	3521
Survcare392	*E. coli*	2019	Rectal /stool swab	0	22	3001
Survcare393	*E. coli*	2019	Nose throat swab	0		3001
Survcare394	*E. coli*	2019	Nose throat swab	1	63	3521
Survcare395	*K. aerogenes*	2019	Groin swab	0	64	3750
Survcare396	*C. freundii*	2019	Anal swab	0	82	3300
Survcare397	*C. koseri*	2019	Rectal /stool swab	1	79	4000
Survcare398	*K. pneumoniae*	2019	Rectal /stool swab	1	79	4000
Survcare399	*K. aerogenes*	2019	Rectal /stool swab	1	14	3001
Survcare400	*E. coli*	2019	Rectal /stool swab	1	71	3001
Survcare401	*Proteus mirabilis*	2019	Rectal /stool swab	1	92	3211

Survcare402	*K. pneumoniae*	2019	Rectal /stool swab	1	64	3820
Survcare403	*E. coli*	2019	Rectal /stool swab	1	64	3820
Survcare404	*K. pneumoniae*	2019	Skin swab	1	56	3620
Survcare405	*C. freundii*	2019	Tracheal secretions	1	71	3001
Survcare406	*K. pneumoniae*	2019	Rectal /stool swab	0	86	3821
Survcare407	*K. pneumoniae*	2019	Rectal /stool swab	0	80	3821
Survcare408	*E. coli*	2019	Urethral Swab	1	38	3000
Survcare409	*E. coli*	2019	Rectal /stool swab	1	62	3430
Survcare410	*C. freundii*	2019	Rectal /stool swab	1	85	3941
Survcare413	*K. pneumoniae*	2019	Nose throat swab	0	70	3941
Survcare414	*Providencia rettgeri*	2019	Urine /Catheter urine	1	84	3941
Survcare415	*K. pneumoniae*	2019	Rectal swab	1	69	3640
Survcare416	*K. pneumoniae*	2019	Rectal swab	0	58	3640
Survcare418	*E. coli*	2019	Nose throat swab	0	59	3941
Survcare419	*K. pneumoniae*	2019	Urine /Catheter urine	0	63	3300
Survcare420	*E. coli*	2019	BAL	1	75	3430
Survcare421	*E. coli*	2019	Rectal /stool swab	0	0	3521
Survcare422	*K. aerogenes*	2019	Rectal /stool swab	0	64	3520
Survcare423	*E. coli*	2019	Rectal /stool swab	1	67	3161
Survcare425	*K. pneumoniae*	2019	Rectal /stool swab	1	78	3750
Survcare426	*E. coli*	2019	Throat swab	0	0	3331
Survcare427	*E. coli*	2019	Wound swab	0	24	3331
Survcare428	*E. cloacae*	2019	Nose throat swab	1	60	3821
Survcare429	*C. freundii*	2019	Rectal /stool swab	1	68	4000
Survcare430	*K. aerogenes*	2019	Rectal /stool swab	0	64	3520
Survcare431	*K. pneumoniae*	2019	Throat swab	1	73	3161
Survcare432	*K. pneumoniae*	2019	Groin swab	1	53	3120
Survcare433	*E. coli*	2019	Rectal swab	1	69	3000
Survcare434	*K. pneumoniae*	2019	Tracheal secretions	1	84	3821
Survcare435	*K. aerogenes*	2019	Rectal /stool swab	1	74	3001
Survcare436	*S. marcescens*	2019	Rectal /stool swab	1	72	3001
Survcare438	*E. coli*	2019	Rectal /stool swab	1	4	3430
Survcare439	*K. pneumoniae*	2019	Urine /Catheter urine	0	83	3300
Survcare440	*K. pneumoniae*	2019	Rectal /stool swab	1	53	3120
Survcare441	*K. pneumoniae*	2019	Rectal /stool swab	0	75	3521
Survcare442	*K. aerogenes*	2019	Rectal /stool swab	0	54	3520
Survcare443	*E. coli*	2019	Rectal /stool swab	0	64	3520
Survcare444	*K. aerogenes*	2019	Rectal /stool swab	1	74	3520
Survcare445	*K. aerogenes*	2019	Rectal /stool swab	0	56	3611
Survcare446	*K. pneumoniae*	2019	Rectal /stool swab	1	76	3102
Survcare447	*E. coli*	2019	Urine /Catheter urine	0	76	3750
Survcare448	*K. aerogenes*	2019	Bronchial secretions/BAL	1	56	4000
Survcare449	*K. pneumoniae*	2019	Decubitus	1	87	3521
Survcare450	*A. baumannii*	2019	Decubitus	1	87	3521
Survcare451	*E. coli*	2019	Urine /Catheter urine	1	75	3820
Survcare452	*A. baumannii*	2019	Groin swab	0	47	3120
Survcare453	*E. coli*	2019	Rectal /stool swab	0	64	3430
Survcare455	*K. pneumoniae*	2019	Urine /Catheter urine	1	68	3750
Survcare457	*C. freundii*	2019	Rectal /stool swab	0	70	3300
Survcare458	*K. pneumoniae*	2019	Rectal /stool swab	1	78	3120
Survcare460	*Raoultella ornithinolytica*	2019	Rectal /stool swab	0	76	3821
Survcare461	*K. pneumoniae*	2019	Rectal /stool swab	0	22	3821
Survcare462	*C. braakii*	2019	Rectal /stool swab	0	64	3821
Survcare463	*E. coli*	2019	Rectal /stool swab	1	59	3001
Survcare464	*K. pneumoniae*	2019	Rectal /stool swab	1	68	3120
Survcare466	*K. pneumoniae*	2019	Urine /Catheter urine	1	91	3000
Survcare467	*K. pneumoniae*	2019	Rectal /stool swab	1	80	3631
ur17031891	*k. pneumoniae*	2017	Urine /Catheter urine	0	52	3770
ur17032073	*K. pneumoniae*	2017	Urine /Catheter urine	0	68	3770
ur17042039	*K. pneumoniae*	2017	Urine /Catheter urine	0	55	3770
ur17046257	*K. pneumoniae*	2017	Urine /Catheter urine	1	82	3770
ur17048087	*K. pneumoniae*	2017	Urine /Catheter urine	0	51	3770
ur18053809	*E. cloacae*	2018	Urine /Catheter urine	1	23	3770
ur18060060	*C. freundii*	2018	Urine /Catheter urine	1	56	3770
ur18060821	*K. aerogenes*	2018	Urine /Catheter urine	0	13	3770
ur18064212	*K. pneumoniae*	2018	Urine /Catheter urine	0	53	3770
ur19075683	*C. freundii*	2019	Urine /Catheter urine	1	78	3770

ur19078501	*K. pneumoniae*	2019	Urine /Catheter urine	0	63	3770
ur19078853	*K. aerogenes*	2019	Urine /Catheter urine	0	54	3770
va17061857	*A. baumannii*	2017	wound Swab	1	76	3770
va17071465	*K. aerogenes*	2017	Rectal /stool swab	1	0	3770
va17079043	*K. pneumoniae*	2017	PEJ swab	0	53	3770
va18094818	*K. variicola*	2018	Vaginal swab	0	79	3770
va18095594	*A. baumannii*	2018	Wound swab	1	57	3770
va18096115	*E. cloacae*	2018	Rectal /stool swab	0	0	3770
va18111651	*Enterobacter xiangfangensis*	2018	Cervical swab	0	20	3770
va19116604	*K. aerogenes*	2019	Rectal /stool swab	0	0	3770
va19138769	*E. cloacae*	2019	Rectal /stool swab	0	0	3770

Supplementary Table S2. Distribution of carbapenemases by species, Hesse, Germany. 2016-2019

Carbapenemase	Species																
	K. pneumoniae	*E. coli*	*A. baumannii*	*C. freundii*	*C. braakii*	*C. koseri*	*C. portucalensis*	*Enterobacter spp.**	*K. michiganensis*	*K. oxytoca*	*K. variicola*	*K. aerogenes*	*Proteus mirabilis*	*P. aeruginosa*	*R. ornithinolytica*	*R. planticola*	*S. marcescens*
**VIM-1**	6			5			1	3	11				3				16
**VIM-2**				3										1			
**VIM-4**				2				1					2	6			1
**NDM-1**	25	2	3	1				1		1							
**NDM-5**	8	16	1	2			1										
**NDM-7**	1																
**KPC-2**	29	21		16		2	1	9			2	2			1	2	
**KPC-3**	41			4			1				1						
**GES-11**			1														
**SHV-38**	3																
**OXA-48**	37	18		13	1			3		1	1				1		2
**OXA-232**	18																
**OXA-244**		25															
**OXA-181**	5	8															
**OXA-143**	1																
**OXA-162**				2					1								4
**OXA-204**		1															
**OXA-23**			36														
**OXA-72**			15														
**OXA-58**			1														
**Total**	174	91	57	48	1	2	4	17	12	2	4	2	5	7	2	2	23

For species: *A. = Acinetobacter. C. = Citrobacter. E. = Escherichia. K. = Klebsiella. P. = Pseudomonas. R. =Raoultella. S. = Serratia*
*. *Enterobacter spp* includes the species *E. cloacae. E. xiangfamngensis* and *E. kobeii.*

Supplementary Table S3: Comparison between 2017, 2018 and 2019 according to bacteria species (x^2-test).

	Species	Year			Significance
		2017	2018	2019	
Carbapenem-resistant (CRGNB)	*A. baumannii*	22	11	21	< 0.01
	K. pneumoniae	51	64	68	n.s.
	E. coli	11	27	68	< 0.001
	C. freundii	7	19	18	n.s.
	Pseudomonas aeruginosa	3	9	1	<0.01
	Enterobacter spp.	5	14	22	n.s.
	K. aerogenes	3	6	27	< 0.01
	other species	24	21	25	n.s.
	Total	126	171	250	
Carbapenemase-producing CRGNB	*A. baumannii*	22	11	21	< 0.05
	K. pneumoniae	42	53	49	n.s.
	E. coli	10	19	58	< 0.001
	C. freundii	7	17	18	n.s.
	Pseudomonas aeruginosa	3	4	1	n.s.
	Enterobacter spp.	1	6	8	n.s.
	K. aerogenes	0	2	0	n.s.
	other species	19	15	18	n.s.
	Total	104	127	173	
Non-Carbapenemase-Producing CRGNB	*K. pneumoniae*	9	11	19	n.s.
	E. coli	1	8	10	n.s.
	C. freundii	0	2	0	n.s.
	Pseudomonas aeruginosa	0	5	0	< 0.01
	Enterobacter spp.	4	8	14	n.s.
	K. aerogenes	3	4	27	< 0.01
	Other species	5	6	7	n.s.
	Total	22	44	77	

Supplementary Table S4: Comparison of carbapenemase proportions among years

	2017		2018		2019		Significance between the years
**VIM**	20	19.0%	18	13.5%	20	11.0%	n.s.
**NDM**	5	4.8%	19	14.3%	36	19.8%	p < 0.01
**KPC**	37	35.2%	38	28.6%	33	18.1%	p <0.05
**OXA-48**	15	14.3%	28	21.1%	33	18.1%	n. s.
**OXA-48-like**	6	5.7%	21	15.8%	38	20.9%	p < 0.01
**OXA-23**	15	14.3%	7	5.3%	13	7.1%	p <0.05
**OXA-72**	7	6.7%	1	0.8%	5	2.7%	p <0.05
**OXA-58**	0		0		1	0.6%	
**GES-11**	0		1	0.8%	0		
**SHV-38**	0		0		3	1.6%	
**Total**	105	100.0%	133	100.0%	182	100.0%	

Supplementary Table S5: Distribution of plasmid Inc types identified

Plasmid Inc type	Plasmid typing	Bacterial Species and Isolate No.																	Total No. of Plasmids
		Kpn	*Eco*	*Cfr*	*Cko*	*Cpo*	*Exi*	*Ecl*	*Kae*	*Kmi*	*Aba*	*Cbr*	*Kox*	*Kva*	*Pmi*	*Ror*	*Rpl*	*Sma*	
		171	91	48	2	4	8	7	2	12	4	1	2	4	3	2	2	23	
N	IncN(ST-5)			2						1									3
	IncN(ST-6)		1																1
	IncN(ST-7)	1		1						7								1	10
	IncN(ST-7)-like			1			1											15	17
	IncN(ST-9)	5																	5
	IncN[ST-15]	23	2	14	2	1	7	3						2		1	2		75
	IncN[ST-15]%	13.5%	2.2%	29.2%	100.0%	25.0%	87.5%	42.9%						50.0%		50.0%	100.0%		
N2	IncN2									3									3
N3	IncN3			2															2
F	IncF[K1:A48*:B-]	3																	3
	IncF[K1:A-:B-]	22								1									23
	IncF[K2:A-:B-]	28		1															29
	IncFIB(K)	51	4	3						4									62
	IncFIB(pQil. AP1918. pB171. (Mar). NDM-Mar)	71	27	3		2		1											104
	IncFIA	13	4																17
	IncFII	7	25	8		1		1								1			43
	IncFIA(HI1)	2																	2
	IncF[A:BC]	2	2						2				1						7
	IncFII(pECLA)						2	1											3
X	IncX1	1	1				2												4
	IncX3	9	13	1															23
	IncX4		4																4
Col	ColRNAI.Col(B512).Col(MG828)	37	14		1														52

	ColpVC	2												2
	ColKPC3	7	2											9
**R**	IncR	43		11										54
**H**	IncHI1A/B	28	1	7				2	1					39
	IncHI2			4		1	6	1						12
**L/M**	IncL/M(pOXA-48)	28	4	1			2	1	2	1	1		5	54
	IncL/M(pMU47)	1	1	1										12
**Q**	IncQ	2	4	3		1						3		13
**Y**	IncY	1	7											8
**I**	IncI1	1	15		1									17
**A/C**	IncA/C2	1		4		1					1			7
**pO111**	pO111		4											4
**B/O/Z**	IncB/O/K/Z		3											3
**TrfA**	TrfA			3			1							4

The species: Aba = *Acinetobacter baumannii*; Cbr = *Citrobacter braakii*; Cfr = *Citrobacter freundii*; Cko= *C. koseri*; Cpo = *C. portucalensis*; Eco = *Escherichia coli*; Ecl = *Enterobacter cloacae* Exi= *Enterobacter xiangfangensis*; Kae =*Klebsiella aerogenes*; Kmi = *Klebsiella michiganensis; Kox= K. oxytoca;* Kpn = *Klebsiella pneumoniae*; Kva = *K. variicola*; Pmi= *Proteus mirabilis*; Ror = *Raoultella ornithinolytica; Rpl = Raoultella planticola; Sma = S. marcescens*.

Supplementary Table S6: The most prevalent plasmid-types of CRGNB isolates in Hesse, Germany, 2016-2019

Plasmid types and No.	Plasmid distribution included in			No. of total isolates harbouring the carbapenemase
	Species	Encoded carbapenemase	No. of plasmid carrying isolates and (%) *	
IncN (pMLST-15); n=75	*K. pneumoniae*	KPC-2	23 (**79**)	29
	E. coli	KPC-2	20 (**95**)	21
	C. freundii	KPC-2	14 (**88**)	16
	Enterobacter spp.	KPC-2	9 (**100**)	9
	Other KPC-2 species **	KPC-2	8 (**80**)	10
IncF[K1:A-:B-]; n=23	*K. pneumoniae*	KPC-3	9 (22)	41
	K. pneumoniae	NDM	8 (24)	34
IncF[K2:A-:B-]; n=29	*K. pneumoniae*	KPC-3	28 (**68**)	41
IncFIB(K); n=62	*K. pneumoniae*	KPC	18 (26)	70
	K. pneumoniae	NDM	15 (44)	34
	K. pneumoniae	OXA-48	12 (32)	37
	K. pneumoniae	OXA-48-like	5 (21)	24
IncFII; n=43	*E. coli*	KPC-2	7 (33)	21
	E. coli	OXA-48-like	16 (47)	34
	E. coli	NDM	2 (11)	18
	K. pneumoniae	NDM	7 (21)	34
	C. freundii	OXA-48	5 (38)	13
IncFIB types: (pQil, AP01918, pB171, (Mar), NDM-Mar) n=104	*K. pneumoniae*	KPC	11 (16)	70
	K. pneumoniae	NDM	28 (**82**)	34
	K. pneumoniae	OXA-48	12 (32)	37
	K. pneumoniae	OXA-48-like	20 (**83**)	24
	E. coli	NDM	12 (**67**)	18
IncX3; n=23	*E. coli*	NDM	6 (33)	18
	E. coli	OXA-48-like	6 (18)	34
IncR; n=54	*K. pneumoniae*	NDM	19 (**56**)	34
	K. pneumoniae	VIM	3 (**50**)	6
IncHI1A/B; n=39	*K. pneumoniae*	NDM	15 (44)	34
	K. pneumoniae.	OXA-48-like	6 (25)	24
IncL/M(pOXA-48); n=54	*K. pneumoniae*	OXA-48	25 (**68**)	37
	C. freundii	OXA-48	8 (**62**)	13
	E. coli	OXA-48	4 (22)	18
	*Other OXA-48 species****	OXA-48	6 (**67**)	9

*, values of ≥ 50% display in bold. **, *C. koseri, C. portucalensis, K.variicola*, *K. aerogenes, R. ornithinolytica* and *R. planticola*. ***, *C. braakii, E. cloacae, E. xiangfangensis, K. varricola, K. oxytoca, R. ornithinolytica and S. marcescens.*

Supplementary Table S7: Prevalence and distribution of the *bla*$_{KPC-2}$-encoding IncN (pMLST15) plasmid

KPC-2-harbouring isolates		*bla*$_{KPC-2}$-encoding IncN(pMLST-15) present in isolates No. and (%)	Percentage distribution in year			
Species	No.		2016	2017	2018	2019
K. pneumoniae	29	23 (79)	16%	11%	26%	47%
E. coli	21	20 (95)	20%	7%	33%	40%
C. freundii	16	14 (88)	21%	29%	29%	21%
Enterobacter spp.	9	9 (100)	0%	13%	50%	38%
*Other species**	10	8 (80)	0%	13%	63%	25%

*, *C. portucalensis, K.variicola*, *K. aerogenes, R. ornithinolytica* and *R. planticola.*

Supplementary Table S8. ST-types of the isolates without any known-carbapenemases (n=154). ND. no MLST scheme available.

Species	ST-type	No. of Isolates per ST
A. pittii (n=1)	Ab. (Pas_804)	1
C. braakii (n=1)	ND	1
C. freundii (n=2)	18	1
	22	1
Cedecea lapagei (n=1)	ND	1
Enterobacter spp. (n=18)	50	2
	68	2
	125	2
	118	2
	57. 81. 89. 165. 170. 177. 466. 672. 837. new	1
E. coli (n=19)	131	5
	405	2
	10. 38. 44. 69. 73. 95. 167. 315. 453. 648. 744. 8130	1
K. aerogenes (n=34)	93 (CC3)	6
	not typable	6
	2. 34 (CC7). 103. 135	1
	New 1 [dnaA_8. fusA_1. gyrB_6. leuS_18. pryG_3. rplB_1. rpoB_2]	1
	New 2[dnaA_10. fusA_3. gyrB_3. leuS_25. pryG-8. rplB_1. rpoB_10]	1
	New 3 [dnaA_2. fusA_3. gyrB_9. leuS_18. pryG-2. rplB_1. rpoB_2]	2
	New 4 [dnaA_6. fusA_2. gyrB_11. leuS_NEW. pryG-4. rplB_3. rpoB_11]	1
	New 5 [dnaA_28. fusA_1. gyrB_6. leuS_18. pryG_3. rplB_1. rpoB_2]	1
	New 6 [dnaA_10. fusA_2. gyrB_3. leuS_14. pryG_2. rplB_1. rpoB_2]	1
	New 7 [dnaA_3. fusA_3. gyrB_2. leuS_9. pryG_2. rplB_1. rpoB_2]	1
	New 8 [dnaA_4. fusA_3. gyrB_6. leuS_27. pryG_2. rplB_1. rpoB_2]	1
	New 9 [dnaA_5. fusA_2. gyrB_9. leuS_5. pryG_6. rplB_0. rpoB_6]	1
	New 10 [dnaA_8. fusA_3. gyrB_1. leuS_3. pryG_2. rplB_1. rpoB_2]	1
	New 11 [dnaA_0. fusA_3. gyrB_9. leuS_18. pryG_2. rplB_0. rpoB_0]	1
	New 12 [dnaA_14. fusA_3. gyrB_6. leuS_27. pryG-2. rplB_1. rpoB_2]	1
	New 13 [dnaA_14. gyrB_10. leuS_29. PryG_3. rplB_8. rpoB_12]	1
	New 14 [dnaA_5. fusA_3. gyrB_1. leuS_25. pryG-8. rplB_1. rpoB_0]	1
	New 15 [dnaA_0. fusA_6. gyrB_0. leuS_15. pryG-7. rplB_1. rpoB_9]	1
	New 16 [dnaA_2. fusA_2. gyrB_0. leuS_3. pryG_2. rplB_1. rpoB_2]	1
	New 17 [dnaA_23. fusA_1. gyrB_16. leuS_14. pryG2. rplB_1. rpoB_2]	1
K. oxytoca (n=11)	2	9
	36. not typable	1
K. pneumoniae (n=41)	45	7
	1626	4
	15	3
	35	3
	48	2
	101	2
	New 1 [gapA_3. infB_3. mdh_2. pgi_1. phoE_10. rpoB_1. tonB_12]	1
	New 2 [gapA_2. infB_1. mdh_1. pgi_2. phoE_10. rpoB_4. tonB_0]	1
	not typable	1
	11. 13. 14. 25. 29. 34. 147. 194. 251. 307. 337. 380. 405. 882. 1727. 1922. 3476	1
K. variicola (n=2)	ND	2
Morganella morganii (n=2)	ND	2
Proteus mirabilis (n=2)	ND	2
Providencia rettgeri (n=1)	ND	1
Pseudomonas aeruginosa (n=6)	175. 274. 348. 395. 792	1
	New ST [acs_16. aro_5. gua_6. mut_11. nuo_4. pps_6. trp_7]	1
Pseudomonas stutzeri (n=1)	ND	1
S. marcescens (n=1	ND	1
**Total**		**154**

Supplementary Table S9. Statistics of ESBL-encoding genes in the isolates without detected carbapenemase

Spezies	No. of CRNGB isolates that without carbapenemase in the genome	Categories of ESBL-encoding genes in the genomes						
		One gene of class C ESBL or Cephalosporin encoding genes (ampC, *bla*$_{\text{ACT}}$, *bla*$_{\text{CMY}}$, *bla*$_{\text{DHA}}$, *bla*$_{\text{OXY}}$, *bla*$_{\text{MIR}}$, *bla*$_{\text{MOR}}$, *bla*$_{\text{PAO}}$)	One gene of class A ESBL (*bla*$_{\text{CTX-M}}$, *bla*$_{\text{TEM-1}}$, *bla*$_{\text{LEN}}$, *bla*$_{\text{SHV}}$) or class D (*bla*$_{\text{OXA-1}}$, *bla*$_{\text{OXA-50}}$, *bla*$_{\text{NPS}}$)	Two genes of class A ESBL (*bla*$_{\text{CTX-M}}$, *bla*$_{\text{TEM-1}}$, *bla*$_{\text{LEN}}$, *bla*$_{\text{SHV}}$) or class D (*bla*$_{\text{OXA-1}}$, *bla*$_{\text{OXA-50}}$, *bla*$_{\text{NPS}}$)	Three or more than three genes of class A ESBL (*bla*$_{\text{CTX-M}}$, *bla*$_{\text{TEM-1}}$, *bla*$_{\text{LEN}}$, *bla*$_{\text{SHV}}$) or class D (*bla*$_{\text{OXA-1}}$, *bla*$_{\text{OXA-50}}$, *bla*$_{\text{NPS}}$)	Class C plus one gene from class A/D	Class C plus two genes from class A/D	none at all
A. pittii	1	1						
C. braakii	1	1						
C. freundii	2						2	
Cedecea lapagei	1							1
E. cloacae	15	13		1				1
E. coli	19	1	10	5		1	1	1
Enterobacter kobei	3							3
Enterobacter xiangfangensis	11	8				2	1	
K. aerogenes	34	1						33
K. oxytoca	11	2				9		
K. pneumoniae	41		10	8	23			
K. variicola	2		1		1			
Morganella morganii	2	2						
Proteus mirabilis	2		2					
Providencia rettgeri	1			1				
Pseudomonas aeruginosa	6					6		
Pseudomonas stutzeri	1							1
S. marcescens	1							1
Total	154	29	23	15	24	18	4	41
Percentage		18.8%	14.9%	9.7%	15.6%	11.7%	2.6%	26.6%

Supplementary Table S10. Epidemiological Statistics from the SurvCARE Hesse Project Dec. 2016 -Dec. 2019

	CRGNB	Distribution per year				Origin				CRGNB from Location			
		before 2017	2017	2018	2019	male	female	Environ-ment	unknown	Hospitals	Medical practices in Hesse	Medical practices barding Hesse	No. of the hospitals
Total isolates submitted	621	42	126	175	278	371	229	12	9	595	17	9	61
Carbapenem-Testing as Non-Resistant (CS)	32	0	0	4	28	19	10	3	0	32	0	0	14
Carbapenem-Testing Resistant Isolates (CR)	589	42	126	171	250	352	219	9	9	563	17	9	61
Carbapenemase-positive CR Isolates	435	31	104	127	173	266	157	8	4	413	15	7	50
% Carbapenemase /CR	74%	74%	83%	74%	69%	76%	72%	89%	44%	73%	88%	78%	
Non-Carbapenemase CR-Isolates	154	11	22	44	77	86	62	1	5	150	2	2	31
% Non-carbapenemase / CR	26%	26%	17%	26%	31%	24%	28%	11%	56%	27%	12%	22%	

Supplementary Table S11. Statistics of genome sequencing and assembly (n=589)

Isolate	Seq-Platform	No. of Reads	Average read length	No. of contigs	N50 (bp)	Genome Size (bp)	GC Content (%)	Coverage
10302a-17	MiSeq	514,362	196.8	229	92.763	5,616,487	57	18.02
10302b-17	MiSeq	671,526	199.8	143	177.013	5,620,835	57	23.87
10304-17	MiSeq	806,028	183	257	92.656	5,624,694	57	26.22
10306-17	MiSeq	2,063,776	141.2	123	291.348	5,571,675	57	52.30
2110128687	MiSeq	1,460,568	198.2	129	154.062	6,519,531	66	44.40
2110129324	MiSeq	1,403,258	202	144	103.646	6,770,223	66	41.87
AF-147492	MiSeq	691,182	203.7	73	226.097	5,013,424	56	28.08
bk17021853	NextSeq	2,603,322	95.6	121	150.755	5,178,170	60	48.06
bk18035705	MiSeq	2,030,010	157.5	52	2.550.858	5,092,383	52	62.79
Entero22	MiSeq	2,405,944	161.2	65	263.287	4,848,013	55	80.00
Entero23	MiSeq	431,008	161.3	516	24.341	4,812,579	55	14.45
NRZ-16960	NextSeq	3,743,664	115.9	176	159.769	5,699,496	57	76.13
NRZ-17079	NextSeq	3,311,630	116.7	232	159.457	5,640,338	57	68.52
NRZ-17685	NextSeq	4,248,906	116.2	145	146.854	5,455,402	57	90.50
NRZ-17710	NextSeq	3,588,144	116.6	169	136.232	5,705,158	57	73.33
NRZ-17768	NextSeq	2,974,538	116.3	187	128.610	5,705,780	57	60.63
NRZ-18377	NextSeq	2,158,094	115.1	183	110.728	5,696,737	57	43.60
NRZ-24454	NextSeq	2,108,306	114.8	141	157.768	5,493,075	57	44.06
NRZ-24773	NextSeq	4,012,020	94.3	500	58.601	5,606,362	57	67.48
NRZ-24797	NextSeq	2,174,144	115.7	127	197.947	5,493,736	57	45.79
NRZ-26443	NextSeq	4,181,718	93.7	343	104.778	5,804,870	57	67.50
NRZ-26447	NextSeq	4,229,744	92.7	1115	12.038	5,592,660	57	70.11
NRZ-27806	NextSeq	3,395,700	92.7	305	66.535	5,488,006	57	57.36
NRZ-28673	NextSeq	8,954,912	115.6	182	224.838	5,800,281	57	178.47
NRZ-29298	NextSeq	4,272,092	116.3	611	26.240	5,729,381	57	86.72
NRZ-29911a	NextSeq	9,252,962	116.1	184	144.367	5,040,339	51	213.13
NRZ-29912	MiSeq	972,598	119.6	379	25.974	4,964,824	52	23.43
NRZ-30046	MiSeq	1,595,192	119.8	212	69.374	5,695,859	57	33.55
NRZ-30817	NextSeq	1,774,382	190.9	160	144.976	5,620,613	57	60.27
NRZ-31125	MiSeq	2,599,996	115.2	314	143.471	5,863,307	57	51.08
NRZ-31131	MiSeq	1,463,586	117	246	50.209	4,967,061	52	34.48
NRZ-31836	NextSeq	4,072,040	132.7	103	189.947	5,184,198	52	104.23
NRZ-32009	NextSeq	6,847,932	36.9	1201	11.550	5,292,600	51	47.74
NRZ-32439	NextSeq	8,394,256	113.1	219	152.121	5,678,641	57	167.19
NRZ-32446	NextSeq	6,670,458	36.9	1422	9.164	5,274,736	51	46.66
NRZ-32813	MiSeq	2,038,760	186.6	217	176.829	7,484,393	65	50.83
NRZ-33225	NextSeq	2,841,946	113.9	103	256.571	5,578,486	57	58.03
NRZ-33411	NextSeq	6,445,722	121.3	367	92.570	5,582,422	50	140.06
NRZ-33607	NextSeq	4,863,758	126	136	215.274	5,423,500	57	113.00
NRZ-33665	NextSeq	8,064,938	36.9	123	99.707	5,299,233	58	56.16
NRZ-33707	NextSeq	3,889,386	140.3	145	214.690	5,567,922	57	98.00
NRZ-33707-1	NextSeq	2,526,378	112.2	156	190.305	5,555,397	57	51.02
NRZ-33707-2	NextSeq	2,129,040	115.6	171	159.041	5,671,690	57	43.39
NRZ-33784	MiSeq	619,978	142.6	3752	9.827	7,291,007	66	12.13
NRZ-33852	NextSeq	4,555,178	134.1	62	274.102	5,465,684	60	111.76
NRZ-33910	NextSeq	4,446,792	94.9	1063	12985	5,108,827	51	82.60
NRZ-33946	NextSeq	2,286,510	126.5	148	105102	5,735,630	57	50.43
NRZ-34952	MiSeq	1,745,912	161.4	260	135.704	7,078,127	66	39.81
NRZ-35373	NextSeq	8,236,456	124.6	151	189.329	5,125,111	51	200.24
NRZ-36109	MiSeq	1,063,064	169.9	323	112.541	6,070,366	56	29.75
NRZ-36387	NextSeq	5,406,518	36.9	4	299	5,058,260	50	39.44
NRZ-36455	MiSeq	683,484	168.7	437	48.831	6,077,753	56	18.97
NRZ-36521	NextSeq	4,445,190	127.5	98	232.010	5,970,510	56	94.93
NRZ-36532	NextSeq	8,517,526	134	56	312.321	5,500,773	60	207.49
NRZ-36825	NextSeq	8,830,950	130.2	104	237.441	5,977,036	56	192.37
NRZ-36826	MiSeq	1,714,212	126	146	123.517	5,983,603	56	36.10
NRZ-36855	NextSeq	8,819,438	125.2	102	168.363	5,554,579	57	198.79
NRZ-37119	NextSeq	7,422,334	113.7	59	692.528	5,077,694	52	166.20
NRZ-37213	MiSeq	2,339,710	168.3	142	215.922	5,667,848	57	69.47
NRZ-37361	MiSeq	1,228,280	122.4	582	52.338	5,625,682	57	26.72
NRZ-37961	NextSeq	12,706,172	123.2	138	194.296	5,776,899	57	270.98
NRZ-37969	NextSeq	9,415,590	36.9	178	89.885	5,183,947	52	67.02
NRZ-38150	NextSeq	4,496,080	127.4	180	165.895	5,720,638	57	100.13

NRZ-38364	NextSeq	5,870,414	115.3	118	179.225	4,925,496	55	137.42
NRZ-38696	NextSeq	13,183,072	124.3	129	292.907	5,591,967	57	293.04
NRZ-38718	MiSeq	886,134	122.6	435	24.473	5,068,436	52	21.43
NRZ-38892	NextSeq	3,026,144	130.5	178	120.228	5,726,678	57	68.96
NRZ-38941	NextSeq	9,044,464	36.9	1212	10.415	5,416,308	57	61.62
NRZ-38954	NextSeq	7,102,658	36.9	1157	11.464	5,442,038	51	48.16
NRZ-39678	NextSeq	8,506,004	121.7	135	224.698	5,783,128	57	179.00
NRZ-39890	MiSeq	1,127,244	147.6	462	30.250	5,170,220	51	32.18
NRZ-40165	NextSeq	9,197,102	124.9	143	161.964	5,708,043	57	201.25
NRZ-40345	MiSeq	1,348,824	133.1	183	167.760	5,589,791	57	32.12
NRZ-40742	NextSeq	4,282,040	125.3	161	199677	5,547,364	57	96.72
NRZ-40869	NextSeq	4,441,332	139.5	155	169.961	5,978,794	56	103.63
NRZ-41277	NextSeq	4,012,860	138.2	215	160.821	5,732,332	57	96.75
NRZ-41473	MiSeq	4,070,042	165.2	74	246.138	6,025,916	56	111.58
NRZ-41623	NextSeq	5,339,138	36.9	1921	5.638	5,323,305	57	37.01
NRZ-41658	MiSeq	996,974	118.6	713	14.119	5,420,388	60	21.81
NRZ-41716	NextSeq	6,177,834	127.1	107	159.034	5,441,434	57	144.30
NRZ-41724	NextSeq	7,849,236	36.9	629	19941	5,464,866	56	53.00
NRZ-42446	NextSeq	14,110,458	116.6	105	249.691	5,845,856	57	281.44
NRZ-42581	NextSeq	21,183,240	116.5	225	202.763	5,603,845	57	440.38
NRZ-43499	NextSeq	1,297,178	120.2	102	168.536	5,640,585	57	27.64
NRZ-43575	NextSeq	10,912,288	111.2	209	173.970	5,095,958	51	238.12
NRZ-45112	NextSeq	10,640,954	115.5	214	105.902	5,782,530	57	212.54
NRZ-45233	NextSeq	3,078,852	140.7	93	192.447	5,503,477	52	78.71
sc17223997	NextSeq	5,563,326	118.9	124	101.188	4,684,932	56	141.19
sc17225761	NextSeq	1,453,664	82.9	450	32.526	5,102,746	50	23.62
sc17236944	NextSeq	7,099,288	114.7	174	118.888	4,798,361	51	169.70
sc17238571	NextSeq	5,443,788	116.6	114	389.978	5,625,113	52	112.84
sc17259839	MiSeq	1,578,002	170.1	119	186.242	5,004,470	55	53.64
sc17262611	NextSeq	17,468,190	114.6	1.200	39.312	6,479,870	56	308.93
sc17266060	NextSeq	4,632,906	113.4	52	280.945	4,734,153	56	110.97
sc17286987	NextSeq	1,918,496	128	311	56.024	5,686,868	52	43.18
sc17288868	MiSeq	1,113,412	148.8	216	93.031	5,592,688	57	29.62
sc17290012	NextSeq	10,017,524	114.1	1.048	57.313	6,662,819	56	171.55
sc17290660	NextSeq	7,421,826	111.9	921	31.244	10,598,852	56	78.36
sc17292733	NextSeq	3,035,802	112.4	113	180.178	5,448,014	57	62.63
sc17294875	NextSeq	3,360,312	117	96	181.108	4,759,989	55	82.60
sc17304042	NextSeq	3,764,254	110.2	542	25.564	4,954,910	51	83.72
sc17312208	MiSeq	1,408,734	176.1	183	194.451	5,664,591	57	43.79
sc17326118	NextSeq	4,838,924	111.5	206	172.927	5,602,578	57	96.30
sc17326834	NextSeq	5,868,128	112.4	208	150.182	5,903,899	57	111.72
sc18328822	MiSeq	955,928	144.9	265	81.182	5,435,638	57	25.48
sc18330209	NextSeq	6,515,320	117.7	85	335.388	5,243,169	58	146.26
sc18353261	NextSeq	3,436,508	125.7	130	194951	5,620,342	57	76.86
sc18364826	MiSeq	1,380,044	212.8	86	181.678	5,078,963	52	57.82
sc18379832	MiSeq	856,794	214.3	56	231.648	5,135,510	55	35.75
sc18383857	MiSeq	1,362,922	199.2	157	111.679	5,664,167	57	47.93
sc18393485	MiSeq	1,118,618	210.9	317	57.021	5,183,975	51	45.51
sc18400719	MiSeq	951,136	198.2	149	97.121	4,811,435	55	39.18
sc18400986	MiSeq	1,277,558	207.3	127	128.771	4,962,012	55	53.37
sc18402389	NextSeq	8,657,486	130.9	80	259.306	4,816,935	55	235.26
sc18407397	NextSeq	18,267,928	119.2	140	337.752	5,237,847	52	415.73
sc18408856	MiSeq	1,229,626	208.2	288	64.479	5,564,517	51	46.01
sc18411631	NextSeq	6,613,800	117.8	281	127.702	5,439,577	50	143.23
sc18412584	NextSeq	5,163,230	134.6	83	487.805	5,100,306	52	136.26
sc18412783	NextSeq	5,509,672	131.5	83	170.962	4,997,670	52	144.97
sc18415124	NextSeq	3,347,162	118.4	95	208.898	5,476,681	57	72.36
sc19427838	NextSeq	4,672,096	121.3	98	173.089	4,801,122	55	118.04
sc19434657	MiSeq	1,595,016	151.9	91	143.489	4,984,550	55	48.61
sc19437368	NextSeq	1,317,446	126.1	248	59.083	5,508,010	57	30.16
sc19437721	NextSeq	3,490,700	116.6	295	51.152	5,157,441	51	78.92
sc19441634	NextSeq	3,393,046	123.1	91	157.291	5,009,360	55	83.38
sc19443197	NextSeq	2,916,868	128.6	62	261.193	5,091,769	55	73.67
sc19443240	NextSeq	3,301,918	127.7	75	175.594	5,130,291	55	82.19
sc19443521-1	NextSeq	2,551,034	129.5	129	102.752	5,481,926	57	60.26
sc19443521-2	NextSeq	3,141,194	128.3	141	108.926	5,480,237	57	73.54
sc19444294	NextSeq	7,167,902	127.6	80	337.489	5,363,202	51	170.54
sc19444656	NextSeq	3,043,190	122.7	66	201.291	5,045,169	55	74.01

sc19445662	NextSeq	3,527,822	123.1	97	189.622	5,208,865	55	83.37
sc19446287	NextSeq	3,308,700	132	136	147.833	5,324,169	52	82.03
sc19460931	NextSeq	1,713,358	134.2	159	118.233	4,809,418	56	47.81
sp17013730	NextSeq	1,872,198	96.8	90	181.277	5,146,821	55	35.21
sp17015437	MiSeq	4,725,040	129	150	177.290	5,598,256	57	108.88
sp17017045	NextSeq	6,389,506	112.9	237	77.729	4,024,797	39	179.23
sp18021199	MiSeq	926,656	206.1	217	61.754	4,884,515	51	39.10
sp19025051	NextSeq	9,352,476	122.1	185	173.189	5,980,920	57	190.93
st17007067	NextSeq	4,513,262	112	130	344.068	5,486,735	57	92.13
Survcare Ex01	MiSeq	2,207,634	170.9	66	370.747	5,461,480	57	69.08
Survcare Ex04	MiSeq	2,022,166	175.1	67	293.934	5,460,406	57	64.85
Survcare Ex05	MiSeq	987,516	169.6	118	133.486	5,457,533	57	30.69
Survcare Ex06	MiSeq	3,078,056	166.4	72	293.326	5,461,782	57	93.78
Survcare Ex07	MiSeq	2,083,380	180.3	75	202.072	5,460,206	57	68.79
Survcare Ex08	MiSeq	1,180,342	165.5	144	125.051	5,457,232	57	35.80
Survcare Ex09	MiSeq	2,595,234	141.4	76	259.811	5,464,249	57	67.16
Survcare Ex10	MiSeq	1,160,486	178	433	48.263	5,520,113	57	37.42
Survcare Ex11	MiSeq	1,696,172	175	183	51.842	3,055,444	38	97.15
Survcare Ex12	MiSeq	2,110,640	172	535	51.229	7,994,827	46	45.41
Survcare002	MiSeq	902,796	158	122	157.028	5,320,157	55	26.81
Survcare003	NextSeq	1,735,154	131.3	222	41.086	3,932,044	39	57.94
Survcare004	NextSeq	1,476,406	129.2	186	39.429	3,862,074	39	49.39
Survcare005	MiSeq	657,938	137.6	983	6.745	3,786,262	40	23.91
Survcare006	NextSeq	1,970,560	130.7	169	63.360	3,936,716	39	65.42
Survcare007	NextSeq	868,840	131.2	233	36.848	3,925,198	39	29.04
Survcare008	MiSeq	1,027,326	108.9	1175	7.808	5,197,049	58	21.53
Survcare009	MiSeq	1,057,930	140.8	345	65.619	5,498,763	57	27.09
Survcare011	MiSeq	1,487,942	143.4	122	159.671	5,461,754	57	39.07
Survcare012	MiSeq	2,343,036	137.4	82	229.859	5,460,636	57	58.96
Survcare013	MiSeq	756,182	141.2	404	35.486	5,613,824	57	19.02
Survcare014	NextSeq	3,828,384	113.2	176	51.392	3,918,042	39	110.61
Survcare015	MiSeq	2,097,340	143.8	184	58.835	3,954,403	39	76.27
Survcare016	MiSeq	2,280,352	132.7	156	50.271	3,874,757	39	78.10
Survcare017	MiSeq	2,490,088	132.2	164	46.410	3,624,415	39	90.83
Survcare018	NextSeq	2,211,116	128.6	164	187.733	5,580,289	57	50.96
Survcare019	MiSeq	3,118,660	164.8	178	61.343	3,953,157	39	130.01
Survcare020	NextSeq	901,224	129.2	192	141.458	5,729,948	57	20.32
Survcare021	NextSeq	1,040,150	124.8	110	227.734	5,454,219	57	23.80
Survcare022	MiSeq	3,452,206	159.9	161	178.006	5,679,381	57	97.20
Survcare023	MiSeq	2,613,582	167.7	146	178.006	5,669,991	57	77.30
Survcare024	NextSeq	8,501,186	122.6	176	179.217	5,622,302	57	185.38
Survcare025	MiSeq	3,991,038	162.4	114	215.428	5,607,471	57	115.59
Survcare027	MiSeq	2,272,446	168.7	266	72.090	5,566,089	57	68.87
Survcare033	MiSeq	5,184,584	245	3420	78.333	6,571,864	57	193.28
Survcare034	MiSeq	1,580,368	183.9	190	197.171	5,598,126	57	51.92
Survcare035	MiSeq	2,171,070	178.3	149	160.456	5,561,535	57	69.60
Survcare036	MiSeq	1,442,396	184.8	216	77.323	5,525,291	52	48.24
Survcare037	MiSeq	2,528,046	174.9	115	292.386	5,564,147	57	79.47
Survcare038	MiSeq	1,383,242	183.8	281	357.278	5,470,863	57	46.47
Survcare039	MiSeq	2,363,814	188.7	115	253.331	5,561,135	57	80.21
Survcare040	MiSeq	2,190,800	189.7	136	292.386	5,573,498	57	74.57
Survcare041	MiSeq	3,387,516	171	204	83.634	5,555,669	57	104.27
Survcare042	MiSeq	1,306,014	185.7	155	214.914	5,674,842	57	42.74
Survcare043	NextSeq	3,410,668	129.6	364	39.448	4,921,761	51	89.81
Survcare044	NextSeq	8,061,708	124.9	117	292.899	5,592,907	57	180.03
Survcare045	NextSeq	10,617,166	120.6	206	118.955	5,061,921	51	252.95
Survcare046	MiSeq	1,859,584	131.5	139	156.833	5,573,180	57	43.88
Survcare047	NextSeq	1,934,410	125.4	120	143703	5,394,322	57	44.97
Survcare048	NextSeq	6,181,780	123.9	187	54505	3,937,882	39	194.50
Survcare049	NextSeq	4,528,358	127	224	46648	3,801,402	39	151.29
Survcare050	NextSeq	2,520,620	122.3	196	90740	5,476,061	52	56.29
Survcare051-1	NextSeq	33,456,170	122.7	123	128483	3,905,106	39	1051.21
Survcare051-2	NextSeq	8,818,982	122.4	122	138925	6,238,858	55	173.02
Survcare052	NextSeq	2,463,384	121.4	335	41022	4,876,519	51	61.33
Survcare053-1	NextSeq	3,154,026	130.1	142	183.679	4,885,701	50	83.99
Survcare053-2	NextSeq	2,707,008	131.4	134	157.768	5,393,753	57	65.95
Survcare053-3	NextSeq	3,685,996	130.4	190	65.120	3,953,500	39	121.58
Survcare055	NextSeq	9,245,592	119.2	176	162182	5,838,485	57	188.76

Survcare056	NextSeq	9,118,568	124	131	185788	5,576,443	57	202.76
Survcare057	NextSeq	4,144,122	127.8	240	44983	3,929,833	39	134.77
Survcare058	NextSeq	4,938,718	125.6	253	81208	5,422,022	50	114.40
Survcare059	NextSeq	6,863,858	126.9	163	60547	3,948,788	39	220.58
Survcare060	NextSeq	5,348,082	120.5	90	207612	5,824,529	55	110.64
Survcare061-1	NextSeq	2,433,626	134.3	63	258.142	5,195,817	60	62.90
Survcare061-2	NextSeq	7,566,490	118.2	130	172.511	6,240,454	55	143.32
Survcare061-3	NextSeq	3,531,640	130.4	156	64.719	3,940,794	39	116.86
Survcare062-1	NextSeq	4,230,530	122.7	243	36984	3,934,684	39	131.93
Survcare062-2	NextSeq	4,821,336	122.6	135	110189	6,235,930	55	94.79
Survcare063-1	NextSeq	9,586,176	117.8	162	162106	5,254,668	51	214.90
Survcare063-2	NextSeq	4,561,460	124	115	167214	6,221,115	55	90.92
Survcare063-3	NextSeq	3,686,066	126.9	212	39636	3,933,601	39	118.91
Survcare064	NextSeq	5,867,882	127.2	141	69992	3,943,841	39	189.26
Survcare065	NextSeq	4,051,772	124.5	112	146294	5,737,718	57	87.92
Survcare066-1	NextSeq	3,901,114	134.1	240	58.399	3,962,430	39	132.02
Survcare066-2	NextSeq	7,388,700	132.8	127	172.512	6,244,570	55	157.13
Survcare067-1	NextSeq	4,115,448	134.6	232	79.721	4,043,242	39	137.00
Survcare067-2	NextSeq	10,165,902	131.8	140	172.511	6,247,125	55	214.48
Survcare068-1	NextSeq	5,007,436	132.3	268	81.672	3,976,400	39	166.60
Survcare068-2	NextSeq	4,806,104	136.2	142	139.626	6,242,040	55	104.87
Survcare069	NextSeq	6,249,946	135	207	162.029	5,367,535	51	157.19
Survcare070	NextSeq	8,122,740	129.3	131	196.617	5,468,421	57	192.06
Survcare071	NextSeq	2,541,750	129.4	176	159.639	5,833,973	57	56.38
Survcare072	MiSeq	965,724	122.9	125	75.469	4,722,599	56	25.13
Survcare074	NextSeq	7,774,630	130.3	132	193.368	6,239,538	55	162.36
Survcare075	NextSeq	3,670,698	129.1	172	172.733	5,770,411	57	82.12
Survcare076	NextSeq	5,405,944	127.4	121	167.921	5,652,477	57	121.84
Survcare077	NextSeq	12,780,800	127.3	308	127.705	5,306,533	50	306.60
Survcare078	NextSeq	9,130,290	131.9	79	339.262	5,595,684	57	215.22
Survcare079	NextSeq	1,551,804	130.7	133	152.739	5,087,877	56	39.86
Survcare080	NextSeq	3,565,214	131.6	120	176.463	5,392,833	51	87.00
Survcare081	NextSeq	7,090,740	135.2	197	162.029	5,366,035	51	178.65
Survcare082	NextSeq	4,144,336	131.8	137	224.625	5,540,674	57	98.58
Survcare083-1	NextSeq	6,404,240	131.3	58	554.586	5,397,907	52	155.78
Survcare083-2	NextSeq	8,726,940	133.3	121	170.936	6,243,296	55	186.33
Survcare084	NextSeq	5,335,888	133.2	50	339.289	4,951,263	55	143.55
Survcare085	NextSeq	6,033,164	130.3	101	271.833	5,903,589	56	133.16
Survcare086	NextSeq	3,333,444	130.2	92	309.694	5,899,211	56	73.57
Survcare087	NextSeq	1,829,196	111.3	304	55715	5,945,049	56	34.25
Survcare088	NextSeq	10,968,008	37.1	146	54.924	3,571,605	39	113.93
Survcare089	NextSeq	8,759,576	37	260	59.204	3,837,812	39	84.45
Survcare090	NextSeq	5,231,946	131.4	107	199.902	5,630,905	57	122.09
Survcare091	NextSeq	2,249,556	134	110	172.696	5,630,857	57	53.53
Survcare092	NextSeq	2,463,614	127.9	124	178.037	5,433,118	51	58.00
Survcare094	NextSeq	29,852,054	131.3	7.591	6.976	13,888,540	51	282.22
Survcare095	NextSeq	3,044,548	126.8	72	174.927	4,814,703	55	80.18
Survcare096	NextSeq	5,394,432	127.8	124	257.085	5,088,661	55	135.48
Survcare097	NextSeq	7,384,570	122.8	208	340.692	5,828,988	57	155.57
Survcare098	NextSeq	2,400,178	130.8	85	235.957	5,557,810	60	56.49
Survcare099	NextSeq	5,695,586	130.9	100	252.062	5,452,648	60	136.73
Survcare100	NextSeq	3,866,532	132.1	84	362.627	5,484,290	60	93.13
Survcare101	NextSeq	2,149,296	135.7	168	147.685	5,495,434	60	53.07
Survcare102	NextSeq	2,010,920	136.1	146	198.717	5,496,652	60	49.79
Survcare103	NextSeq	7,974,940	130.9	150	196.633	4,964,319	51	210.28
Survcare104	MiSeq	1,405,376	119.6	277	63.380	5,168,185	51	32.52
Survcare105	NextSeq	7,149,388	130.6	108	194.074	5,697,853	57	163.87
Survcare107	NextSeq	5,011,040	136	151	204.097	5,838,166	57	116.73
Survcare108	NextSeq	4,415,662	141.4	122	160.067	5,531,255	57	112.88
Survcare109	NextSeq	4,598,572	139.7	84	224.838	5,645,308	57	113.80
Survcare111	NextSeq	11,150,536	110.5	174	127.020	4,949,359	51	248.95
Survcare112	NextSeq	7,073,898	116.7	133	94.554	4,059,052	39	203.38
Survcare113	NextSeq	4,806,358	116.6	104	206.727	5,534,585	57	101.26
Survcare114	NextSeq	6,363,410	110.3	237	92.111	5,052,215	51	138.93
Survcare115	NextSeq	10,161,314	110.1	63	266.521	5,292,337	57	211.39
Survcare116	NextSeq	13,050,280	107.9	115	131.000	4,031,985	39	349.24
Survcare117	NextSeq	3,008,816	115.1	106	168.372	5,633,487	57	61.47
Survcare118	NextSeq	6,514,080	114.6	96	224.698	5,646,272	57	132.21

Survcare119	NextSeq	5,530,364	111.5	212	186.237	5,164,971	51	119.39
Survcare120	NextSeq	3,185,962	110	70	338.383	5,223,944	52	67.09
Survcare121	NextSeq	2,938,752	116.2	261	137.651	5,130,710	51	66.56
Survcare122	NextSeq	3,020,072	109	297	29.044	3,980,818	39	82.69
Survcare123	NextSeq	4,378,956	117.7	69	308.863	5,292,403	57	97.39
Survcare124	NextSeq	2,131,340	114.9	131	148.378	5,968,605	56	41.03
Survcare125	NextSeq	6,050,376	117.2	169	127.484	5,117,072	51	138.58
Survcare126	NextSeq	6,485,398	113.7	95	232.010	5,971,960	56	123.48
Survcare127	NextSeq	6,563,592	110.1	93	247.566	5,469,172	57	132.13
Survcare128	NextSeq	1,285,380	115.4	550	43.440	7,158,010	66	20.72
Survcare129	NextSeq	5,270,092	120.9	164	189.353	5,153,173	51	123.64
Survcare130	NextSeq	3,745,620	125	172	202.976	5,139,277	50	91.10
Survcare131	NextSeq	3,955,794	121.4	127	211.368	5,534,711	57	86.77
Survcare132	NextSeq	7,832,216	121.2	128	350.592	5,659,887	52	167.72
Survcare133	NextSeq	3,187,948	123.9	301	125.873	5,467,566	50	72.24
Survcare134	NextSeq	7,617,892	118	302	156.278	5,466,835	50	164.43
Survcare135	NextSeq	8,505,492	122.9	78	165.416	4,923,801	55	212.30
Survcare137	MiSeq	2,345,034	175.2	124	138.095	5,450,346	52	75.38
Survcare138	MiSeq	2,136,648	184.9	289	37.686	4,015,773	39	98.38
Survcare139	MiSeq	2,229,038	168.2	149	225.774	5,627,476	57	66.62
Survcare140	MiSeq	2,265,428	183.5	322	72.216	5,448,824	50	76.29
Survcare141	MiSeq	3,122,824	183.6	193	59.063	3,950,452	39	145.14
Survcare142	MiSeq	2,446,182	169.5	223	92.489	5,558,488	57	74.59
Survcare143	NextSeq	19,690,492	108	81	205.902	3,981,468	39	534.12
Survcare145	NextSeq	2,580,542	129.8	77	214.767	5,483,226	57	61.09
Survcare146	NextSeq	19,391,424	115.7	107	207.369	4,976,146	56	450.87
Survcare148	NextSeq	19,883,942	105.8	184	68.020	3,938,978	39	534.08
Survcare149	NextSeq	13,610,724	119.4	112	167.921	5,423,196	57	299.66
Survcare150	NextSeq	10,238,604	114.8	120	199.532	5,614,486	52	209.35
Survcare151	NextSeq	23,300,668	117.3	123	164.585	5,121,795	55	533.63
Survcare153	NextSeq	2,949,394	106.6	333	186.894	5,627,897	57	55.87
Survcare154	NextSeq	1,160,096	120	117	122.785	5,530,281	52	25.17
Survcare155	NextSeq	657,484	123.7	442	28.874	6,443,423	66	12.62
Survcare156	NextSeq	1,055,296	123.8	247	73.315	6,352,057	66	20.57
Survcare157	NextSeq	28,562,266	121.9	199	269.086	7,232,790	66	481.38
Survcare158	NextSeq	919,840	124.1	391	49.015	6,977,073	66	16.36
Survcare159	NextSeq	994,592	123.5	332	47.821	7,168,148	66	17.14
Survcare160	NextSeq	1,423,218	116.4	212	136.630	5,412,987	51	30.60
Survcare161	NextSeq	1,030,790	115.3	146	101.906	5,210,629	52	22.81
Survcare162	NextSeq	917,716	115.4	326	34.672	5,159,728	52	20.53
Survcare163	NextSeq	859,732	114.4	371	48.045	5,278,687	52	18.63
Survcare164	NextSeq	4,865,812	128.6	197	190.925	5,767,812	57	108.49
Survcare165	NextSeq	24,852,458	118.2	94	195.364	5,042,749	52	582.53
Survcare166	NextSeq	821,410	119.4	196	50.194	3,862,262	51	25.39
Survcare167	NextSeq	5,370,528	133.3	56	417.474	5,119,897	55	139.83
Survcare168	NextSeq	3,947,104	132.9	162	199.572	5,356,250	50	97.94
Survcare169	NextSeq	3,826,278	125.4	93	259.143	5,001,313	55	95.94
Survcare170	NextSeq	3,335,372	131.9	146	206.734	5,728,559	57	76.80
Survcare171	NextSeq	4,830,566	131.4	98	277.756	5,885,869	57	107.84
Survcare172	NextSeq	3,782,526	128.9	172	156.425	5,833,371	57	83.58
Survcare173	NextSeq	4,187,742	130.3	145	120.883	5,575,511	57	97.87
Survcare174	MiSeq	907,558	208.5	59	259.750	5,210,558	59	36.32
Survcare175	NextSeq	1,025,834	140.2	62	158.682	4,219,570	63	34.08
Survcare176	NextSeq	2,589,952	140.4	194	88.879	4,922,062	51	73.88
Survcare177	NextSeq	1,571,260	137.8	180	96.019	7,084,563	66	30.56
Survcare178	NextSeq	2,607,220	140.2	139	112.372	4,122,071	39	88.68
Survcare179	NextSeq	2,026,090	138.7	77	221.395	6,035,851	55	46.56
Survcare180	NextSeq	2,424,154	140.3	59	376.760	5,176,880	55	65.70
Survcare182	NextSeq	2,404,242	138.4	179	118.514	7,157,405	66	46.49
Survcare184	NextSeq	1,814,642	139	191	137.078	5,195,047	51	48.55
Survcare185	NextSeq	5,844,798	140.3	171	145.794	3,951,131	39	207.54
Survcare186	NextSeq	3,789,336	140.5	135	186.523	5,712,471	57	93.20
Survcare188	NextSeq	4,361,332	135.9	114	242.797	5,425,346	52	109.25
Survcare189	NextSeq	4,142,752	138.8	66	311.219	5,297,973	58	108.53
Survcare190	NextSeq	3,875,484	138.5	157	112.685	5,011,718	51	107.10
Survcare191	NextSeq	5,002,160	137.7	89	371.798	5,482,339	57	125.64
Survcare192	NextSeq	2,746,024	138.5	62	312.263	5,073,067	55	74.97
Survcare193	NextSeq	5,205,192	139	66	478.392	5,109,183	55	141.61

Survcare194	NextSeq	4,444,194	138.4	58	266.521	5,290,629	57	116.26
Survcare202	NextSeq	4,142,638	138.6	155	203.127	5,658,411	57	101.47
Survcare203	NextSeq	6,267,086	141.1	50	435.635	4,880,284	55	181.20
Survcare204	NextSeq	1,330,952	95.7	283	44.804	5,698,213	57	22.35
Survcare212	MiSeq	2,672,466	149.4	112	145.631	5,247,939	51	76.08
Survcare213	MiSeq	1,489,710	175	147	91.886	4,084,312	39	63.83
Survcare217	MiSeq	1,759,344	204.5	96	232.179	5,645,982	57	63.72
Survcare218	MiSeq	1,373,574	202.7	114	201.907	5,043,894	55	55.20
Survcare219	MiSeq	3,158,072	163.3	54	540.639	4,870,558	55	105.88
Survcare221	MiSeq	1,190,006	212.2	154	125.274	5,217,523	51	48.40
Survcare222	MiSeq	1,311,652	202.3	110	312.791	5,622,145	57	47.20
Survcare223	MiSeq	1,702,836	221.3	134	95.230	4,265,548	39	88.34
Survcare224	MiSeq	969,970	202	90	222.487	5,185,414	55	37.79
Survcare225	MiSeq	747,730	195.6	1.014	74.618	5,959,543	55	24.54
Survcare226	MiSeq	645,686	198.4	116	200.095	5,754,815	57	22.26
Survcare227	MiSeq	907,496	196.2	251	70.761	5,542,927	60	32.12
Survcare228	MiSeq	599,344	176.2	1339	15.046	5,597,024	60	18.87
Survcare229	MiSeq	617,922	156.8	143	148.366	5,296,158	57	18.29
Survcare230	MiSeq	1,869,374	209.8	133	112.231	4,919,647	51	79.72
Survcare231	MiSeq	1,244,268	195	131	202.174	6,017,589	55	40.32
Survcare232	MiSeq	1,417,938	150.5	138	85.858	5,032,107	55	42.41
Survcare233	MiSeq	921,242	161.1	271	67.404	5,141,269	55	28.87
Survcare234	MiSeq	1,677,634	151	124	92.206	4,953,094	51	51.14
Survcare235	MiSeq	1,412,266	161.3	117	109.131	5,083,010	52	44.82
Survcare236	MiSeq	928,482	153	311	37.270	5,234,213	51	27.14
Survcare237	MiSeq	706,138	164.8	156	63.294	4,991,440	55	23.31
Survcare238-2	MiSeq	5,595,558	162.9	74	285.992	5,411,707	60	168.43
Survcare239	MiSeq	4,161,136	162.1	123	167.872	5,369,257	52	125.63
Survcare240	MiSeq	836,614	175.1	42	275.180	4,750,787	55	30.84
Survcare241	MiSeq	828,826	168.3	655	49.609	5,629,412	57	24.78
Survcare242	MiSeq	905,690	140.8	113	151.324	5,153,293	52	24.75
Survcare243	MiSeq	539,396	146.7	938	10.423	5,695,279	57	13.89
Survcare244	MiSeq	1,125,552	134.7	205	84.526	5,040,348	51	30.08
Survcare245	NextSeq	7,166,522	117.7	55	385.403	5,087,199	55	165.81
Survcare246	MiSeq	2,303,730	167.5	30	480.397	5,141,068	55	75.06
Survcare247	NextSeq	4,307,898	108.7	90	189.762	5,293,350	52	88.46
Survcare248	MiSeq	760,446	136.8	645	21.581	5,718,381	57	18.19
Survcare249	NextSeq	3,802,712	118.6	91	252.359	5,377,103	57	83.87
Survcare250	NextSeq	6,872,492	112.1	69	215.808	5,316,763	58	144.90
Survcare251	NextSeq	6,501,486	116.6	105	224.846	5,637,256	57	134.48
Survcare252	NextSeq	3,717,604	121.7	126	156.774	5,301,727	52	85.34
Survcare253	NextSeq	5,251,570	119	168	117.002	4,971,544	50	125.70
Survcare254	NextSeq	5,504,330	122.4	161	137.697	4,960,625	51	135.82
Survcare255	NextSeq	59,077,304	115.8	108	125.195	3,922,568	39	1744.05
Survcare257	MiSeq	2,688,166	157.8	75	509.146	4,996,488	55	84.90
Survcare258	MiSeq	2,786,204	137.5	62	252.162	5,571,474	60	68.76
Survcare260	MiSeq	3,246,446	140.1	106	260.652	5,896,112	57	77.14
Survcare261	MiSeq	751,236	156.9	272	47.386	5,522,963	57	21.34
Survcare262	MiSeq	938,690	133.6	220	62.479	4,918,891	51	25.50
Survcare263	MiSeq	1,439,654	108.2	202	70.919	5,439,327	57	28.64
Survcare265	NextSeq	4,498,428	120.5	143	190.962	5,312,570	50	102.03
Survcare266	NextSeq	5,048,254	120.9	133	91.424	4,042,748	39	150.97
Survcare267	NextSeq	2,033,270	122.2	254	68.281	5,978,483	57	41.56
Survcare268	NextSeq	3,549,292	128	179	93.227	5,560,981	60	81.70
Survcare269	NextSeq	4,205,496	122.2	162	203.915	5,563,142	57	92.38
Survcare270	MiSeq	1,454,156	183.3	72	326.858	5,559,400	60	47.95
Survcare271	MiSeq	1,310,254	167.6	154	187.925	5,714,246	57	38.43
Survcare272	MiSeq	2,453,906	168.9	1.516	4.837	4,103,050	39	101.01
Survcare273	MiSeq	1,103,816	180.3	96	263.868	5,526,274	57	36.01
Survcare274	NextSeq	4,381,874	126	88	242.045	5,078,010	55	108.73
Survcare275	NextSeq	4,707,476	121	166	184.946	5,202,394	51	109.49
Survcare276	NextSeq	2,037,332	116.3	176	119.479	6,175,172	56	38.37
Survcare277	NextSeq	3,071,588	117.6	206	49.022	3,927,916	39	91.96
Survcare278	NextSeq	2,283,182	116.1	231	136.365	5,208,442	51	50.89
Survcare279	MiSeq	3,446,736	146.1	81	324.586	5,508,951	57	91.41
Survcare280	MiSeq	2,060,466	154	124	214.923	5,820,021	57	54.52
Survcare281	MiSeq	1,000,234	131.3	1.067	8.613	5,410,393	60	24.27
Survcare282	MiSeq	1,665,222	173.3	426	24.406	5,549,337	59	52.00

Survcare283	NextSeq	2,067,738	109	161	146.463	5,211,036	55	43.25
Survcare284	MiSeq	1,172,158	162.1	182	86.990	5,585,287	57	34.02
Survcare285	MiSeq	1,749,976	146.3	274	35.806	3,996,847	39	64.06
Survcare286	MiSeq	1,871,322	72.7	3.464	1.483	4,361,159	59	31.19
Survcare287	NextSeq	1,681,212	113.7	223	140.379	5,713,980	57	33.45
Survcare289	NextSeq	7,375,080	120.1	293	84.669	4,960,673	51	178.55
Survcare290	MiSeq	2,175,158	172	145	133.172	5,532,075	57	67.63
Survcare291	NextSeq	1,846,328	123	137	184.607	5,163,778	51	43.98
Survcare292	NextSeq	5,780,280	112.8	255	88.243	4,742,519	51	137.48
Survcare293	NextSeq	2,331,126	118.8	294	82.967	4,958,838	51	55.85
Survcare294	NextSeq	1,110,338	110.6	722	24.846	5,422,171	60	22.65
Survcare295	MiSeq	3,202,730	167.9	154	150.947	5,264,577	51	102.14
Survcare296	MiSeq	3,180,024	163.1	79	209.265	4,903,378	55	105.78
Survcare298	MiSeq	4,546,540	144	2896	4.222	4,098,411	51	159.75
Survcare299	MiSeq	600,712	156.1	765	10.584	3,980,092	39	23.56
Survcare300	NextSeq	5,970,586	103.8	109	264.402	5,428,329	57	114.17
Survcare302	MiSeq	1,787,122	154.4	160	229.820	6,485,396	55	42.55
Survcare303	MiSeq	1,024,636	145	438	61.536	5,532,675	50	26.85
Survcare306	MiSeq	539,460	162.3	581	19.524	5,278,427	52	16.59
Survcare308	MiSeq	3,444,594	160.1	162	274.738	5,125,867	52	107.59
Survcare309	MiSeq	1,593,056	144.6	253	78.932	4,977,600	51	46.28
Survcare310	MiSeq	534,638	157.9	637	15.478	4,868,215	51	17.34
Survcare311	NextSeq	3,324,406	110	93	279.542	5,382,572	52	67.94
Survcare312	MiSeq	10,686,084	146.5	43	372.310	5,082,945	58	307.99
Survcare313	MiSeq	1,300,020	171	187	137.236	5,292,618	51	42.00
Survcare314	NextSeq	758,506	110.4	1.013	10.802	5,587,804	52	14.99
Survcare315	MiSeq	734,976	180.9	102	182.471	5,247,961	52	25.34
Survcare316	MiSeq	684,730	165.4	105	212.466	5,159,069	52	21.95
Survcare317	MiSeq	794,108	161.1	3092	6.068	5,389,919	51	23.74
Survcare318	MiSeq	1,004,484	154	378	53.487	5,432,278	51	28.48
Survcare319	NextSeq	2,796,540	102.5	277	124.399	5,269,567	51	54.40
Survcare320	NextSeq	2,830,270	113.8	108	246.078	5,278,951	52	61.01
Survcare321	NextSeq	2,865,788	115.4	232	89.525	5,270,104	51	62.75
Survcare323	NextSeq	4,911,550	115.2	121	79.906	3,957,686	39	142.96
Survcare324	NextSeq	2,466,180	117.2	128	74.411	3,957,434	39	73.04
Survcare325	NextSeq	5,879,626	112.6	97	192.746	5,090,138	55	130.06
Survcare326	NextSeq	30,581,464	114.7	63	197.645	5,416,999	55	647.53
Survcare327	NextSeq	6,757,306	123.2	171	160.582	5,126,649	51	162.39
Survcare328	NextSeq	2,913,840	124.4	767	41.656	5,859,510	57	61.86
Survcare329	NextSeq	5,418,292	120.3	285	66.242	4,720,594	51	138.08
Survcare330	NextSeq	7,681,486	108.6	244	58.674	4,106,334	39	203.15
Survcare331	NextSeq	10,164,258	116.6	147	110.154	4,109,588	39	288.39
Survcare332	NextSeq	7,203,032	115.5	184	62.095	4,093,939	39	203.22
Survcare333	NextSeq	4,931,206	119.5	453	69.450	5,840,379	57	100.90
Survcare334	NextSeq	1,653,162	118	150	132.506	5,157,795	50	37.82
Survcare335	NextSeq	2,925,024	109.3	158	131.400	5,024,677	51	63.63
Survcare336	NextSeq	3,033,486	106.8	47	423.121	4,940,785	52	65.57
Survcare337	NextSeq	4,851,124	120.6	99	231.615	4,854,123	51	120.53
Survcare338	NextSeq	1,849,822	121.4	846	23.503	5,616,398	57	39.98
Survcare339	NextSeq	2,163,188	123	277	69.165	5,162,487	55	51.54
Survcare340	NextSeq	3,925,124	119.6	112	89.673	3,849,646	39	121.94
Survcare341	NextSeq	3,015,996	120.9	293	125.157	5,226,451	51	69.77
Survcare342	NextSeq	3,546,852	124.2	115	230.806	5,548,295	54	79.40
Survcare343	NextSeq	6,024,030	120.6	129	206.734	5,811,324	57	125.01
Survcare344	NextSeq	6,366,526	123	91	383.265	4,041,266	39	193.77
Survcare346	NextSeq	7,109,396	115.6	77	197.549	4,730,036	55	173.75
Survcare347	NextSeq	6,452,720	123	65	425.812	4,884,842	54	162.48
Survcare348	NextSeq	11,509,338	119.7	134	200.379	5,276,690	54	261.09
Survcare349	NextSeq	12,192,764	120.5	47	264.574	5,226,709	57	281.10
Survcare350	NextSeq	20,992,508	116.8	197	100.549	4,991,000	50	491.27
Survcare351	NextSeq	13,145,438	118.8	119	206.734	5,809,921	57	268.80
Survcare353	NextSeq	10,605,316	119.8	76	371.566	5,260,563	57	241.52
Survcare354	NextSeq	1,968,868	122.7	129	173.296	5,439,105	57	44.42
Survcare355	NextSeq	4,409,928	118.9	106	187.848	5,087,656	55	103.06
Survcare356	NextSeq	5,988,132	114.8	821	8.763	3,845,423	39	178.77
Survcare357	NextSeq	5,388,486	110.2	156	96.391	4,120,473	39	144.11
Survcare358	NextSeq	3,734,172	119.5	81	185.268	4,833,000	55	92.33
Survcare359	NextSeq	4,167,076	118.1	148	76.669	3,935,491	39	125.05

Survcare360	NextSeq	5,598,504	115	269	76.214	5,325,505	51	120.90
Survcare361	NextSeq	5,886,920	118.8	100	200.258	5,487,276	57	127.45
Survcare362	NextSeq	4,543,680	117.6	85	210.194	4,993,080	55	107.02
Survcare364	NextSeq	7,890,970	115.3	116	135.574	5,529,902	57	164.53
Survcare365	NextSeq	955,860	110.6	930	13.335	6,252,830	56	16.91
Survcare366	MiSeq	1,359,952	113.6	1486	46.608	5,186,643	56	29.79
Survcare367	MiSeq	1,477,476	98.6	4.967	3520	6,676,383	57	21.82
Survcare368	MiSeq	1,937,802	115.5	147	142.733	5,599,022	57	39.97
Survcare369	MiSeq	1,794,260	134.4	2610	20.345	6,317,855	55	38.17
Survcare370	MiSeq	764,380	128.6	99	119.304	5,244,190	55	18.74
Survcare371	MiSeq	2,874,124	121.7	443	81.425	6,324,015	56	55.31
Survcare372	MiSeq	1,043,666	102.3	1116	19.125	4,430,641		24.10
Survcare374	NextSeq	3,903,744	107.3	64	187.442	5,273,857	58	79.42
Survcare375	NextSeq	2,477,256	111	80	198.395	5,281,484	58	52.06
Survcare376	NextSeq	12,073,400	110.2	161	165.137	5,865,870	57	226.82
Survcare377	NextSeq	9,070,610	110.9	292	121.488	5,412,883	50	185.84
Survcare378	NextSeq	1,634,628	109.4	1.358	34.353	5,524,302	50	32.37
Survcare379	NextSeq	1,848,980	110.7	238	91.607	5,233,761	51	39.11
Survcare380	NextSeq	2,662,436	134	234	111.430	5,285,307	51	67.50
Survcare381	NextSeq	2,231,436	124.3	272	90.279	5,281,724	51	52.51
Survcare382	NextSeq	13,380,742	108.3	221	147.763	5,332,687	51	271.75
Survcare383	NextSeq	1,802,912	112.1	246	119.461	5,330,655	51	37.91
Survcare384	NextSeq	3,856,620	111.8	228	127.596	5,333,493	51	80.84
Survcare385	NextSeq	3,373,474	109.7	215	84.027	5,025,853	51	73.63
Survcare386	NextSeq	25,930,246	104.4	356	133.644	5,094,315	51	531.40
Survcare387	NextSeq	5,173,262	101.9	263	77.036	5,115,084	51	103.06
Survcare388	NextSeq	2,096,308	103.4	340	76.816	5,137,987	51	42.19
Survcare389	NextSeq	2,255,918	104.4	293	97.899	5,331,359	51	44.18
Survcare390	NextSeq	4,197,158	102.6	554	32.820	5,368,386	51	80.22
Survcare391	NextSeq	2,583,346	110.6	310	22.220	3,762,547	39	75.94
Survcare392	NextSeq	4,870,888	109.4	346	118.828	5,469,110	50	97.43
Survcare393	NextSeq	2,375,434	110.4	394	121.488	5,469,305	50	47.95
Survcare394	NextSeq	3,963,376	107.7	219	120.920	5,126,120	50	83.27
Survcare395	NextSeq	1,705,080	104.5	136	81.648	5,048,019	55	35.30
Survcare396	NextSeq	1,202,112	111.3	213	89.634	5,384,741	52	24.85
Survcare397	NextSeq	1,753,432	110	84	168.380	4,850,364	54	39.77
Survcare398	NextSeq	1,684,540	107.7	210	73.110	5,498,051	57	33.00
Survcare399	NextSeq	5,517,782	131.8	77	220.586	5,139,048	55	141.51
Survcare400	NextSeq	3,713,808	128.8	275	133.826	5,011,467	51	95.45
Survcare401	NextSeq	1,306,352	115.6	204	78.298	4,254,255	39	35.50
Survcare402	NextSeq	1,348,668	131.2	133	130.709	5,511,746	57	32.10
Survcare403	NextSeq	1,320,882	133.9	150	134.210	4,902,609	51	36.08
Survcare404	NextSeq	1,624,962	132.3	145	113.343	5,434,635	57	39.56
Survcare405	NextSeq	2,511,860	127.6	274	54.597	5,195,100	52	61.70
Survcare406	NextSeq	2,872,074	127.9	141	152.189	5,661,880	57	64.88
Survcare407	NextSeq	2,728,472	131.7	125	222.263	5,659,447	57	63.49
Survcare408	NextSeq	1,535,748	120.9	189	97.062	4,897,660	51	37.91
Survcare409	NextSeq	1,469,962	132.3	322	64.115	5,425,199	51	35.85
Survcare410	NextSeq	2,653,664	124.3	111	126.305	5,137,615	52	64.20
Survcare413	NextSeq	1,542,274	131.2	125	127.702	5,313,424	57	38.08
Survcare414	NextSeq	1,898,430	130.2	587	16.632	4,302,776	41	57.45
Survcare415	NextSeq	2,234,866	130.7	189	173.491	5,585,289	57	52.30
Survcare416	NextSeq	2,508,312	128.7	188	126.468	5,557,075	57	58.09
Survcare418	NextSeq	2,556,516	128	243	99.841	5,264,881	51	62.15
Survcare419	NextSeq	2,450,388	131.3	145	159.137	5,804,875	57	55.43
Survcare420	NextSeq	2,913,940	125.7	268	52.117	5,006,209	51	73.17
Survcare421	NextSeq	4,114,892	121.4	294	111.188	5,533,616	50	90.28
Survcare422	NextSeq	2,931,456	129	107	179.661	5,050,060	55	74.88
Survcare423	NextSeq	2,749,750	127.8	195	122.332	5,145,463	51	68.30
Survcare425	NextSeq	11,427,594	123.1	103	225.563	5,571,869	57	252.47
Survcare426	NextSeq	3,242,792	128.2	175	190.507	5,094,801	50	81.60
Survcare427	NextSeq	1,976,738	132.7	142	262.710	5,087,638	50	51.56
Survcare428	NextSeq	2,948,850	131.9	74	286.889	5,169,030	55	75.25
Survcare429	NextSeq	1,325,928	130.2	117	166.880	5,289,103	52	32.64
Survcare430	NextSeq	1,391,032	134.4	84	320.569	5,050,122	55	37.02
Survcare431	NextSeq	2,719,494	112.1	1.391	8.134	5,666,098	57	53.80
Survcare432	NextSeq	3,832,876	121.9	101	211.750	5,536,694	57	84.39
Survcare433	NextSeq	4,050,090	75.3	3.670	1.661	4,157,826	53	73.35

Survcare434	NextSeq	4,068,424	119.4	78	195.687	5,514,053	57	88.10
Survcare435	NextSeq	6,573,962	122.4	63	210.241	5,183,645	55	155.23
Survcare436	NextSeq	7,076,292	115.2	75	252.062	5,604,794	60	145.44
Survcare438	NextSeq	9,738,120	121.4	287	133.904	5,443,253	50	217.19
Survcare439	NextSeq	6,506,788	121.3	129	206.734	5,809,332	57	135.86
Survcare440	NextSeq	3,865,254	122.2	95	199.384	5,526,473	57	85.47
Survcare441	NextSeq	3,573,572	126.7	125	174.473	5,537,088	57	81.77
Survcare442	NextSeq	1,976,072	129.2	91	221.566	5,047,021	55	50.59
Survcare443	NextSeq	4,506,884	122.4	290	52.381	5,109,777	51	107.96
Survcare444	NextSeq	2,541,066	127.8	73	210.167	5,181,839	55	62.67
Survcare445	NextSeq	2,276,584	132.4	57	243.708	5,106,894	55	59.02
Survcare446	NextSeq	3,280,830	134	286	119.373	6,010,584	57	73.14
Survcare447	NextSeq	3,692,274	132.3	170	167.898	5,332,476	51	91.61
Survcare448	NextSeq	2,136,164	126.6	79	334.758	5,253,646	55	51.48
Survcare449	NextSeq	3,007,522	129.1	130	172.762	5,589,112	57	69.47
Survcare450	NextSeq	3,348,548	119.7	182	46.351	4,005,752	39	100.06
Survcare451	NextSeq	2,799,162	119.3	203	89.644	5,125,090	51	65.16
Survcare452	NextSeq	4,584,100	110	159	94.905	3,974,454	39	126.87
Survcare453	NextSeq	3,837,984	121.7	199	133.487	4,959,552	51	94.18
Survcare455	NextSeq	7,662,936	118.6	187	214.690	5,914,740	57	153.65
Survcare457	NextSeq	7,395,134	117.8	124	236.617	5,382,929	51	161.84
Survcare458	NextSeq	7,172,644	121.4	88	202.161	5,446,746	57	159.87
Survcare460	NextSeq	6,735,610	124	59	293.341	5,616,560	56	148.71
Survcare461	NextSeq	14,202,912	124.1	159	250.021	5,804,360	57	303.67
Survcare462	NextSeq	5,890,684	116.6	59	281.554	4,920,770	52	139.58
Survcare463	NextSeq	8,319,712	121	272	119.366	5,216,046	51	193.00
Survcare464	NextSeq	7,732,298	116.1	91	255.159	5,453,380	57	164.62
Survcare466	NextSeq	15,763,942	106.3	101	203.828	5,520,904	57	303.52
Survcare467	NextSeq	7,612,358	110.5	164	168.645	5,943,993	57	141.52
ur17031891	NextSeq	7,083,278	114.5	94	214.957	5,318,045	57	152.51
ur17032073	NextSeq	5,189,384	111.1	133	152.649	5,593,037	57	103.08
ur17042039	NextSeq	14,813,708	113.4	1.499	25.553	6,517,075	57	257.77
ur17046257	NextSeq	7,972,230	109.4	211	81.059	5,568,776	57	156.62
ur17048087	NextSeq	4,849,034	105	151	182.174	5,598,724	57	90.94
ur18053809	NextSeq	3,281,400	115.6	61	301.794	4,683,439	56	80.99
ur18060060	NextSeq	4,269,056	110.1	145	316.593	5,523,667	52	85.09
ur18060821	MiSeq	799,046	213.6	101	130.422	5,153,327	55	33.12
ur18064212	MiSeq	797,748	212.8	172	87.759	5,437,298	57	31.22
ur19075683	NextSeq	5,846,848	122.9	137	118.396	5,102,860	52	140.82
ur19078501	NextSeq	9,240,882	124.2	112	266.435	5,771,393	57	198.86
ur19078853	NextSeq	10,854,164	128.5	82	225.778	5,156,656	55	270.48
va17061857	NextSeq	3,340,984	113.5	132	91.011	4,177,710	39	90.77
va17071465	NextSeq	10,357,040	111.7	296	200.240	10,319,657	54	112.10
va17079043	NextSeq	2,510,270	113.8	205	143.677	5,660,867	57	50.46
va18094818	NextSeq	14,670,582	116.3	77	320.686	5,588,288	57	305.32
va18095594	NextSeq	2,918,660	129.8	297	29790	3,907,578	39	96.95
va18096115	NextSeq	7,130,396	125	131	91.423	4,892,662	55	182.17
va18111651	NextSeq	10,938,948	130.7	119	301.482	5,306,949	55	269.41
va19116604	NextSeq	5,526,432	117.8	72	251.322	5,115,562	55	127.26
va19138769	NextSeq	2,266,602	133.1	65	203.388	4,467,498	56	67.53

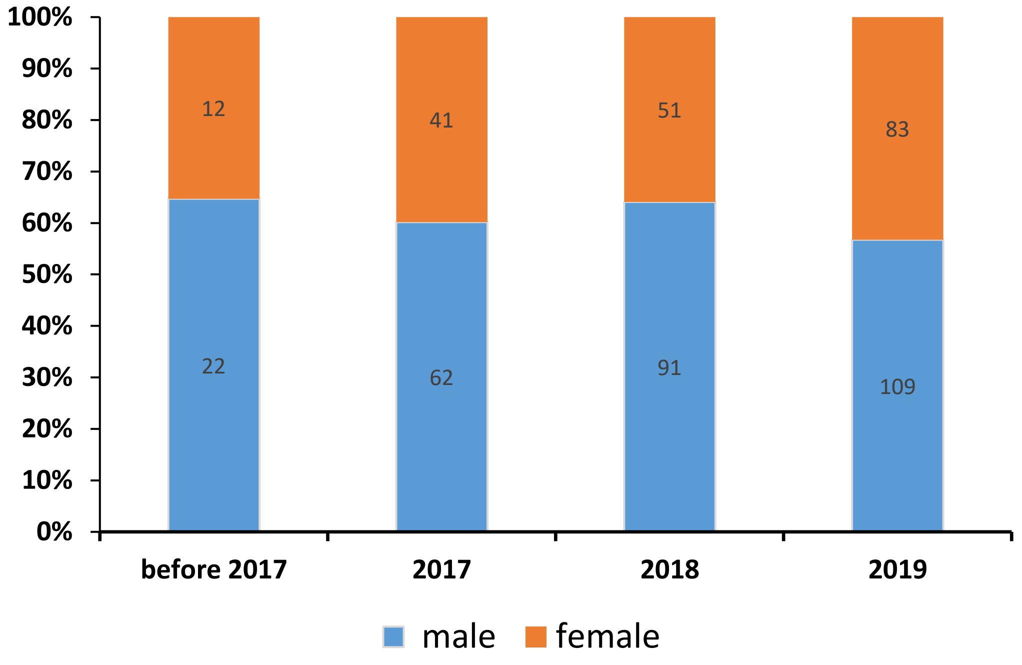

Supplementary Figure S1. The CRGNB carrier distribution according to the patient gender during the SurvCARE study

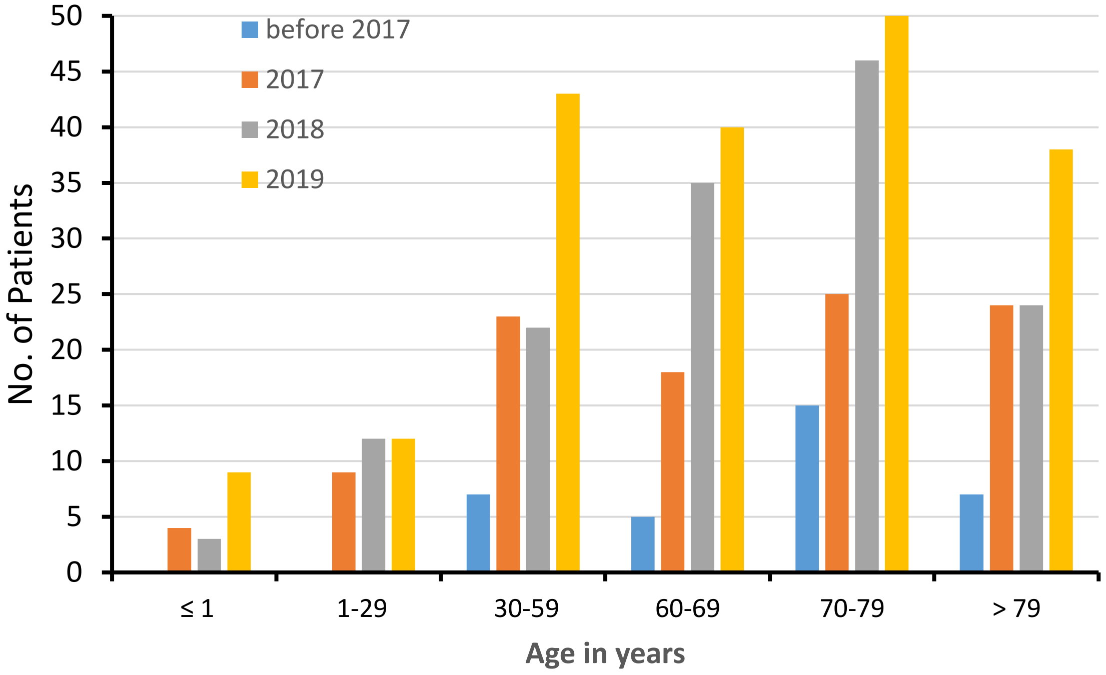

Supplementary Figure S2. The CRGNB carrier distribution according to the patient age groups during the SurvCAREstudy

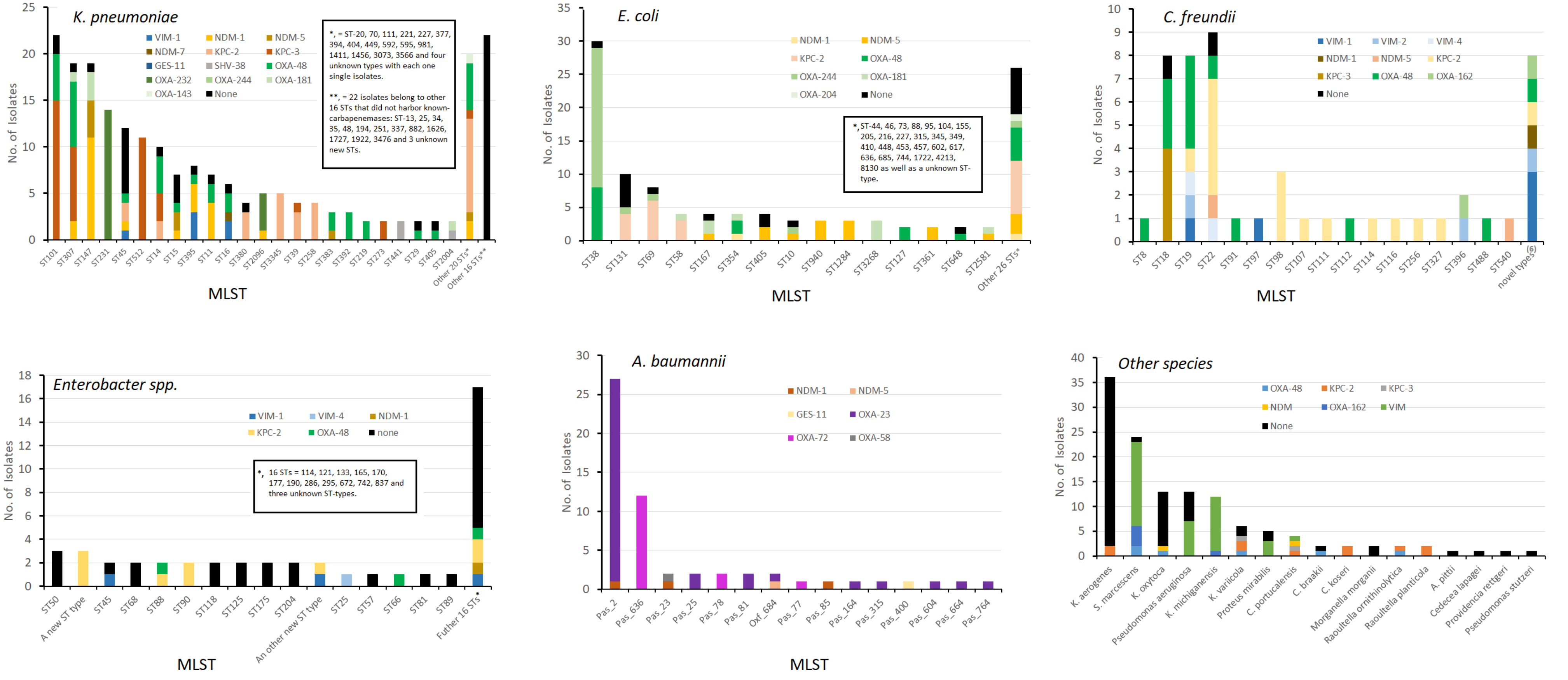

Supplementary Figure S3: Distribution of identified carbapenemase types in *K. pneumoniae, E. coli, C. freundii, Enterobacter spp.* and *A. baumannii*, as well as other species, according to the sequence types. *Enterobacter spp*. includes isolates of *E. cloacae, E. kobei* and *E. xiangfangensis*

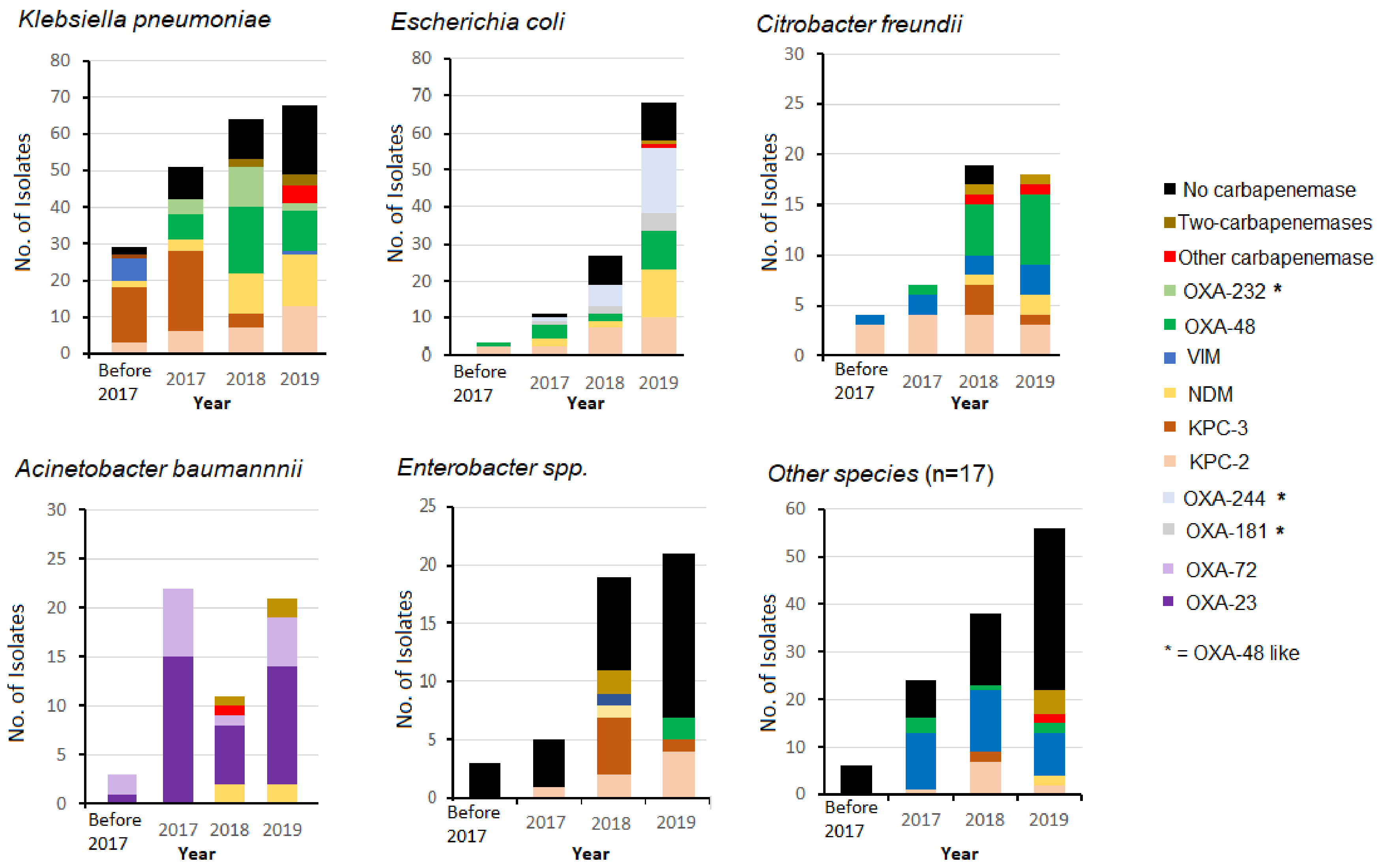

Supplementary Figure S4. Distribution of carbapenemase types within the predominant species *Klebsiella pneumoniae, Escherichia coli, Citrobacter freundii, Acinetobacter baumannii*, *Enterobacter spp.* and other species (details on STs are shown in Figure S4) within the study period.

Isolates	Species	ST-type	VIM-1	VIM-4	KPC-2	OXA-48	OXA-162	NDM-5	OXA-181	SHV-38	OXA-23	NDM-1	OXA-232	OXA-58	Source	Sex	Year
1	*C. freundii*	ST22													Rectal swab /stool	M	2017
2	*E. xiangfangensis*	ST88													Cervical swab	F	2018
3	*E. cloacae*	Unknown													Wound swab	F	2018
4	*C. freundii*	ST44													Urine / catheter-Urine	M	2018
5	*K. pneumoniae*	ST147													Anal swab	M	2018
6	*K. pneumoniae*	ST147													Rectal swab /stool	M	2018
7	*A. baumannii*	ST218 *													Urine Catheter swab	F	2018
8	*Proteus mirabilis*	ND													Groin swab	F	2019
9	*Proteus mirabilis*	ND													Rectal swab /stool	M	2019
10	*S. marcescens*	ND													Rectal swab /stool	M	2019
11	*S. marcescens*	ND													Rectal swab /stool	F	2019
12	*S. marcescens*	ND													Rectal swab /stool	M	2019
13	*K. pneumoniae*	ST388													Urine / catheter-Urine	M	2019
14	*E. coli*	ST2851													Urine / catheter-Urine	F	2019
15	*K. pneumoniae*	ST2004													Rectal swab /stool	F	2019
16	*A. baumannii*	ST684 *													Rectal swab /stool	M	2019
17	*K. pneumoniae*	ST2096													Tracheal secretions	F	2019
18	*A. baumannii*	ST642 *													Rectal swab /stool	M	2019

Supplementary Figure S5. Isolates carrying two types of carbapenemases. ND: MLST scheme not available (*. according to the Oxford *Acinetobacter baumannii* MLST Schema)

Cluster No.	Species	MLST	Carbapenemase-Gene	Other common beta-lactamase-genes	No. of Isolates
1	*Kpn*	14	OXA-48	TEM-1	4
2	*Kpn*	14	KPC-3	SHV-1	3
3	*Kpn*	39	KPC-2	TEM-1	3
4	*Kpn*	101	KPC-3	SHV-1	15
5	*Kpn*	101	OXA-48	SHV-1, TEM-1A	5
6	*Kpn*	147	NDM-1		11
7	*Kpn*	147	OXA-181	SHV-11, TEM-1	3
8	*Kpn*	147	NDM-5		4
9	*Kpn*	231	OXA-232	CTX-M-1, TEM-1	14
10	*Kpn*	258	KPC-2	SHV-11	4
11	*Kpn*	307	KPC-3	CTX-M-15, SHV-28	8
12	*Kpn*	307	OXA-48	CTX-M-14b, SHV-28	7
13	*Kpn*	380	KPC-2	SHV-1, TEM-1	3
14	*Kpn*	392	OXA-48	CTX-M-15, SHV-11, TEM-1	3
15	*Kpn*	395	VIM-1	CTX-M-15, SHV-11	3
16	*Kpn*	395	NDM-1	SHV-11	3
17	*Kpn*	512	KPC-3	SHV-11	11
18	*Kpn*	2096	OXA-232	CTX-M-15, SHV-28, TEM-1A	3
19	*Kpn*	3345	KPC-2	SHV-27, TEM-1	5
20	*Eco*	38	OXA-244	CTX-M-27/14	19
21	*Eco*	38	OXA-48	CTX-M-24/14, TEM-1	8
22	*Eco*	69	KPC-2	TEM-1	6
23	*Eco*	58	KPC-2	TEM-1	3
24	*Eco*	1284	NDM-5		3
25	*Eco*	3268	OXA-181	CTX-M-15, TEM-1B	3
26	*Cfr*	18	KPC-2	CMY-84, OXA-1, TEM-1	3
27	*Cfr*	18	KPC-3	CMY-16, CMY-79, OXA-10	4
28	*Cfr*	18	OXA-48	CMY-79, TEM-1	3
29	*Cfr*	19	OXA-48	CMY-41	3
30	*Cfr*	22	KPC-2	CMY-48, TEM-1	4
31	*Exi*	new*	KPC-2	ACT-15, CTX_M-9, TEM-1	3
32	*Kmi*	213	VIM-1	OXY-1-3	6
33	*Kmi*	205	VIM-1	OXY-1-1	3
34	*Pmi*		VIM-1 + VIM-4	CTX-M-15	3
35	*Sma*		VIM-1	SRT-2	16
36	*Sma*		VIM-1 + OXA-162		3
37	*Aba*	2	OXA-23	ADC-25	26
38	*Aba*	636	OXA-72	ADC-25	12

Cluster No.	Districts and Hospital ID																																							
			KA							GM						WL						FO											DA							FB	
	0	0a	1	2	3	4	20	31	32	6	27	28	29	30	38	5	14	16	22	23	36	7	10	11	13	15	17	21	24	33	34	35	8	9	12	18	25	26	37	19	39
1								■																■							■										
2	■																■																					■			
3													■														■									■					
4							■																					■												■	
5													■				■										■											■			
6																	■	■								■	■									■					
7																						■	■													■					
8																						■	■	■																	
9										■											■				■				■												
10										■												■					■														
11																	■							■												■	■	■			
12	■																					■													■						
13																																									■
14																	■										■									■					
15										■																															
16			■																			■					■														
17																		■	■	■																■					
18			■																		■								■												
19									■								■															■					■				
20			■							■			■									■			■						■							■	■		
21			■							■		■										■														■					
22																									■		■									■					
23											■																									■	■				
24																									■										■						
25																												■									■				
26																						■														■					
27																						■																			
28	■	■																																							
29														■															■												
30																													■							■					
31																	■																			■					
32			■	■	■	■																																			
33																■																									
34																						■											■	■							
35			■																																						
36			■																																						
37													■					■				■			■		■									■		■			
38															■							■					■			■											

Supplementary Figure S6: Characteristics of clusters of carbapenemase-producing Gram-negative bacteria. Districts: DA = Darmstadt; FB = Fulda -Bad Hersfeld; FO = Frankfurt-Offenbach; GM = Giessen-Marburg; KA = Kassel; WL= Wiesbaden-Limburg. Medical practices within and outside Hesse are noted by "0" and "0a". Bacterial Species *Aba = Acinetobacter baumannii*; *Cfr = Citrobacter freundii*; *Eco = Escherichia coli*, *Exi= Enterobacter xiangfangensis*; *Kpn= Klebsiella pneumoniae*; *Kmi = Klebsiella michiganensis*; *Pmi =Proteus mirabilis*; *Sma = Serratia marcescens*. New*, these harbored identical alleles of MLST-genes for *Enterobacter cloacae*, *dnaA*_51, *fusA*_23, *gyrB*_4, *leuS*_6, *pyrG*_~117, *rplB*_4 and *rpoB*_6.

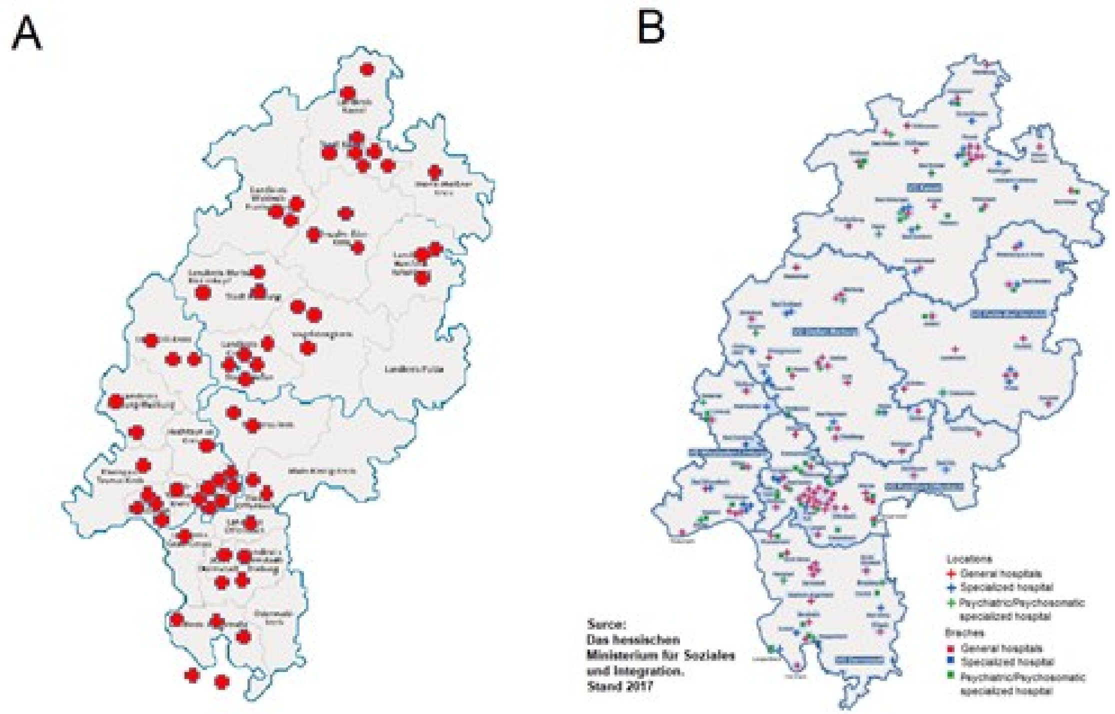

Supplementary Figure S7. The locations of the participating hospitals in the SurvCARE study (A) and locations of all hospitals in Hesse (B), Germany.
